# A dynamic multi-tissue model to study human metabolism

**DOI:** 10.1038/s41540-020-00159-1

**Published:** 2021-01-22

**Authors:** Patricia Martins Conde, Thomas Pfau, Maria Pires Pacheco, Thomas Sauter

**Affiliations:** 1 Department of Life Sciences and Medicine, University of Luxembourg, Esch-sur-Alzette, Luxembourg; 2 Megeno S.A., Esch-sur-Alzette, Luxembourg

**Keywords:** Biochemical networks, Multicellular systems, Computer modelling, Biomarkers, Dynamic networks

## Abstract

Metabolic modeling enables the study of human metabolism in healthy and in diseased conditions, e.g., the prediction of new drug targets and biomarkers for metabolic diseases. To accurately describe blood and urine metabolite dynamics, the integration of multiple metabolically active tissues is necessary. We developed a dynamic multi-tissue model, which recapitulates key properties of human metabolism at the molecular and physiological level based on the integration of transcriptomics data. It enables the simulation of the dynamics of intra-cellular and extra-cellular metabolites at the genome scale. The predictive capacity of the model is shown through the accurate simulation of different healthy conditions (i.e., during fasting, while consuming meals or during exercise), and the prediction of biomarkers for a set of Inborn Errors of Metabolism with a precision of 83%. This novel approach is useful to prioritize new biomarkers for many metabolic diseases, as well as for the integration of various types of personal omics data, towards the personalized analysis of blood and urine metabolites.

## Introduction

Genome scale reconstructions of the human metabolism have become ever more sophisticated in the recent years (EHMN^1^, Recon1-3^2–5^, and HMR1-2^6,7^). Multi-tissue models based on these reconstructions, or from scratch^8,9^, are used to investigate the interplay between different tissues. And while there are profiles for uptake, and release of substances from individual cell lines (e.g., Jain et al.^10^), this information is often lacking for human tissues, and has to be inferred from other species. Thus, it is difficult to properly constrain a multi-tissue model, as literature sources vary, and bounds might be too stringent. Recently a study by Hyötyläinen et al.^11^ suggested to relax these hard constraints by using a quadratic programming approach in which hard bounds are replaced by penalties for data violation. This approach enables the direct use of data in a model, while contradicting bounds are relaxed in an automatic fashion, with many smaller violations preferred to large individual violations. However, this approach still does not address another issue with multi-tissue models of higher organisms. What is the metabolic objective of the organism? For microbes, biomass accumulation has been extensively used as objective yielding good results^12^, and this has also shown promising results for plants^13^, and even infant growth^14^. This objective is also commonly used for cancerous tissues which grow rapidly^15^. However, for a mature organism that no longer grows, this objective is questionable. Other suggested options include enzymatic optimization, or energetic optimization, the former trying to minimize the overall fluxes, the latter trying to minimize the use of ATP^16^. But while efficiency is a reasonable target, it is likely not to be the major driving force in an organism. In human, one observable factor is that blood metabolite levels are tightly regulated^17^, and metabolism is likely to play an important role in keeping these levels steady. Abnormal levels of metabolites commonly indicate diseases, e.g., Diabetes Mellitus and Inborn Errors of Metabolism (IEMs). Temporary perturbations of metabolite levels occur primarily due to nutrient uptake or food scarcity. This would imply that a good objective drives metabolite levels towards the healthy range after perturbations by nutrient uptake and simultaneously tries to keep metabolite levels stable while fasting.

In this paper, we introduce a novel framework for multi-tissue modeling that allows to incorporate blood metabolite levels and flux measures of tissue uptake/secretion. We validated our model, first by predicting the effect of temporary perturbations, i.e., long duration fasting, nutrient uptake and also by simulating exercise at different intensity levels. We further applied stronger perturbations such as the knock-out of genes-associated with IEMs to investigate their impact on blood metabolites levels, and on urine excretion rates. 90% of the investigated metabolic changes occurring during incremental exercise could be matched with previous data, and the prediction of blood amino acids biomarkers had a precision of 83%.

## Results

### Description of the multi-tissue model

**Fig. 1:**
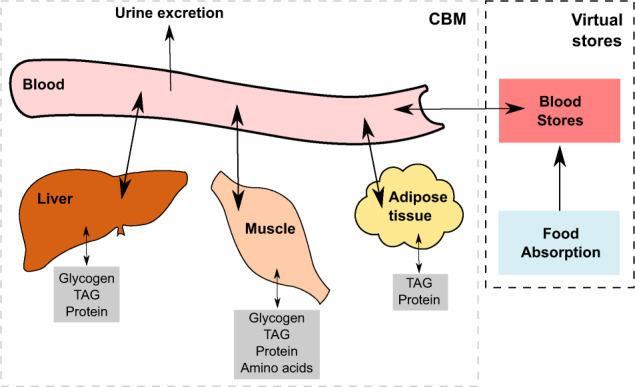
Model overview. The multi-tissue model is divided in two parts. The first part, denoted here as CBM, contains the three tissue models, the blood compartment, and the urine excretion reactions. The second part, denoted here as virtual stores, contains the blood stores which are updated at each time step. The internal stores of each tissue are represented in grey with the stored compounds named in the boxes. The food absorption mimics the food absorbed by the gut and transferred to the blood. TAG = Triacylglycerol.

In this work a dynamic multi-tissue model able to predict human metabolic activities in different healthy and unhealthy conditions was developed. To achieve this, three constraint-based metabolic tissue models (Fig. 1), liver, muscle and adipose tissue were reconstructed from Recon2.04^3^ and tissue specific transcriptomic data, and integrated to create a multi-tissue model that contains a total of 7251 reactions, and 5311 metabolites. After reconstructing each individual tissue model using the FASTCORMICS workflow (see Methods), each one was evaluated independently to determine how many known tissue-specific functions each model was able to perform, as defined in Gille et al.^18^. Two examples of tested functions are: (1) the ability to produce ATP from glucose or a fatty acid, in presence or absence of oxygen; (2) the ability to degrade an amino acid to ammonium. In total, the liver model could perform 139 out of the 142 tested functions, the muscle model 88 out of the 98 functions, and the adipose tissue model 91 out of the 98 tested functions. To conclude, each model was able to perform a minimum of 90% of the known tissue specific functions (Supplementary Data 1), indicating the high quality of the reconstructions.

In the following step, the different tissue models were coupled. Each model was thereby individually connected with its own storage compartment containing glycogen, triacylglycerol (TAG), protein and amino acids stores, if applicable, and with the blood compartment. The latter contains a storage for the different blood metabolites, thus allowing for the description of the dynamics of these metabolites. Each of these stores were initialized with the average amounts generally found in healthy individuals, as described in the Methods section. To mimic food absorption from the gut to the blood, a previously established differential equation^19^, which has been used to simulate glucose absorption, from the gut to the blood, was integrated in the workflow of the multi-tissue model simulation. In this work, this equation was used to simulate the gut to blood absorption of all meal metabolites which was achieved by optimizing the parameters for each of the meal metabolites of interest.

In this work, a dynamic Flux Balance Analysis (dFBA)^20^ based approach was employed to simulate the final coupled model. In contrast to the general FBA approach, where the metabolic flux can be calculated for a given specific time point and condition, the dFBA approach enables to account for the dynamics of metabolites by integrating the individual FBA solution over time. In both settings, constraints on reaction fluxes, and the optimization of an objective function are usually employed to narrow down the solution space and to obtain biological relevant flux solutions, respectively. The model in this paper represents the dynamics of metabolism of three interconnected tissue models, and to properly simulate it, an objective function, integrating multiple sub-objectives, has been developed as follows: (1) blood homeostasis should be maintained; (2) energy ingested in excess may be stored in the tissue energy stores, and may be used in periods of food scarcity; and (3) metabolic transitions are smooth over time, and between conditions. For a more detailed description of the model simulation workflow, and the objective function, please refer to the Methods section.

A second evaluation on the coupled multi-tissue model was performed. The second set of tests consisted of simulating different physiological conditions and a set of IEMs, and validating the model predictions using published data. In the next sections, the model predictions will be presented and discussed thoroughly.

### Effect of fasting, ingestion of a low and a high fat meal at the metabolic level

After setting up the model, we validated it through the simulation of different metabolic conditions occurring throughout the day. I.e. in the morning when we wake up we are in a fasting state, and after having breakfast the metabolism needs to adapt to a fed condition. Thus, these states were simulated and the ability of metabolism adaption between conditions was verified.

**Fig. 2:**
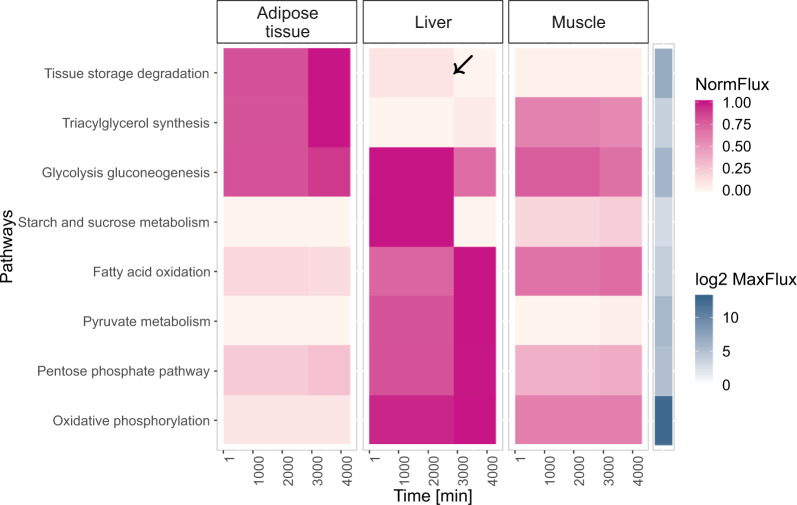
Metabolic pathways activity in different tissues associated with energy metabolism during fasting. The pathway *tissue storage degradation* contains different reactions depending on each tissue. In the adipose tissue, this pathway contains the reaction that leads to the degradation of TAG from the adipose tissue stores. In the muscle, and in the liver, this pathway corresponds to the degradation of glycogen from the glycogen stores, and to the degradation of TAG from the fat stores in each tissue. The black arrow, occurring at around 2880 min, represents the point when the liver glycogen stores were depleted, leading to a switch in the metabolic activity. Each flux in a pathway was normalized to the maximum flux of that pathway for all tissues.

We started by simulating a fasting condition for 3 days, which corresponds to 4320 min, to determine if the model was able to capture known effects occurring during the fasting state, such as liver glycogen depletion. As can be seen in Fig. 2, the three tissues activated different energy-associated metabolic pathways during the fasting condition. At around 2 days of fasting (2880 min), a metabolic switch was apparent. This shift corresponds to the point where glycogen stores were completely depleted in the liver tissue. The *starch and sucrose metabolism* flux was at the maximum in the liver, but once the glycogen stores became depleted it became inactive. This pathway contains all the reactions involved in either the production or hydrolysis of glycogen. The effect was further confirmed by the flux through the *glycolysis/gluconeogenesis* pathway, and through the *tissue storage degradation pathway*. The latter one, in the liver, contains the reaction which leads to the degradation of the glycogen stores. These two pathways followed a similar pattern as the *starch and sucrose metabolism*. They became fully inactive after the glycogen stores were depleted. To compensate for this effect, and to fulfill the energy demands of the multi-tissue model, there was an increase in the flux through the *tissue storage degradation*, and the *triacylglycerol synthesis* pathways in the adipose tissue. The *triacylglycerol synthesis* pathway contains reactions producing and degrading TAG. In the liver, the flux through the *glycolysis/gluconeogenesis* pathway was decreased, while the flux through the *fatty acid oxidation* increased. To conclude, the model predicted that after 2 days of prolonged fasting, the liver glycogen stores were completely depleted, which led to an increase of TAG hydrolysis rate in the adipose tissue.

**Fig. 3:**
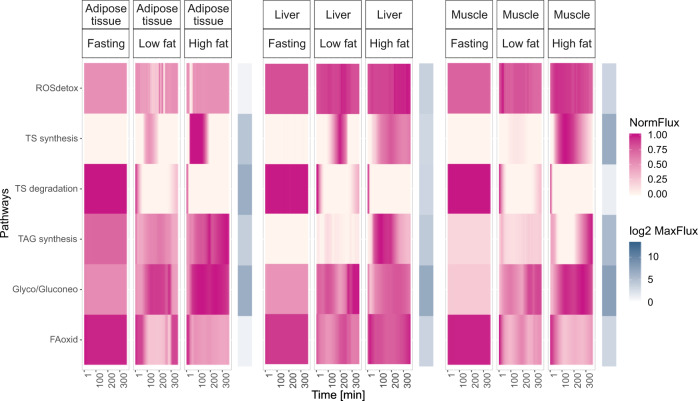
Comparison of the effect of different conditions on the energy associated pathways in different tissues. The *tissue storage degradation* contains different reactions depending on the tissue. In the adipose tissue, this pathway contains the reaction that leads to the degradation of TAG from adipose tissue stores. In the muscle, and in the liver, this pathway corresponds to the degradation of glycogen from the glycogen stores, and to the degradation of TAG from the fat stores in each tissue. The *tissue storage synthesis* contains different reactions depending on each tissue. In the adipose tissue, this pathway contains the reaction that leads to the TAG synthesis, and its storage in the adipose tissue. In the muscle, and in the liver, this pathway corresponds to the synthesis, and storage of glycogen, and TAG in each tissue. Each flux in a pathway was normalized to the maximum flux of that pathway for each tissue. The fluxes were normalized in this way, as no striking pathway flux dynamics could be observed in the adipose tissue when the normalization was performed to the maximum value of a pathway among all tissues. ROSdetox = Reactive oxygen species (ROS) detoxification; TS synthesis = Tissue storage synthesis; TS degradation = Tissue storage degradation; TAG Synt = Triacylglycerol synthesis; Glyco/Gluconeo = Glycolysis, and gluconeogenesis; FAoxid = Fatty acid oxidation.

Throughout the day, the metabolism goes through different metabolic states: from fasting, usually in the morning, to feeding, after ingesting a meal, and depending on the individual’s lifestyle, the type of meal selected will be different. Thus, we decided to compare and determine the metabolic effect of ingesting two meals with different fat content^21^. These two meals, representing a healthy and a high fat breakfast, were compared to each other and to the fasting condition (Fig. 3 and Supplementary Fig. 1). Thus, three different conditions were simulated for 6 h (360 min): a fasting condition, a low and a high fat meal consumption. When comparing the fluxes of all tissues through all the metabolic pathways in the three contrasting conditions, the results suggest that the adipose tissue was metabolically less active than the liver and muscle in all cases (Supplementary Fig. 1). This result was expected as this tissue has a lower ATP demand, when compared to the liver and the muscle. In addition, the adipose tissue appeared to be quite unaffected by the different tested conditions, possibly because the pathway fluxes in the adipose tissue were much smaller when compared to the other two tissues. The model predicted that the liver tissue was the main amino acid metabolizer, as the flux through the amino acids metabolic pathways was larger in liver. There were two exceptions: the aromatic amino acids and the glycine, serine, alanine and threonine metabolism, which were mainly active in the muscle (Supplementary Fig. 2).

To further investigate the effect of the three simulated conditions (fasting, low and high fat meal) on the energy-associated metabolic pathways, the fluxes were normalized to the maximum flux per pathway, and per tissue (Fig. 3).

When compared to the fasting condition, the fed state led to the activation (e.g., *glycolysis/gluconeogenesis*), or inactivation (e.g., *fatty acid oxidation*) of metabolic pathways, which as the post-absorptive state progressed, slowly returned to the fasting flux values. The effect of the meals on the majority of the energy-associated pathways was still highly pronounced 6 h after the meal ingestion.

In the fed state, the *glycolysis/gluconeogenesis* pathway became the main source of energy in the three tissues, and the TAG entering the adipose tissue was stored, and not used as an energy source. Concomitantly, the *fatty acid oxidation* was decreased in the fed state.

The *ROS detoxification* pathway was more active in the liver and in the muscle during all the conditions, and the high fat meal led to a further increase of *ROS detoxification* in these tissues.

The results correctly suggest that the liver was the main metabolizer of amino acids^22^. Furthermore, during the fasting state, *fatty acid oxidation* was increased in all tissues. In the fed state, the *fatty acid oxidation* rate decreased while the *glycolysis/gluconeogenesis* rate increased, and this metabolic adaptation has already been previously reported^23–25^. In addition, in this condition, the degradation of the internal energy did not occur, instead energy compounds derived from the meal (e.g., TAG and glucose) were used and the surplus was stored in the internal energy stores. The activation of these metabolic pathways, following a meal, are in good agreement with known human metabolism^22^. In addition, the ingestion of a high fat meal led to an increase of *ROS detoxification* in the liver, and to a higher degree in the muscle. It has already been shown that meals rich in carbohydrates and lipids lead to the increased production of ROS^26,27^, which has been associated with the development of insulin resistance^27,28^. These results confirm the potential detrimental effect that high fat diets might have on muscle and liver metabolism, and show the value of the availability of such a modeling approach.

As shown in Supplementary Fig. 3, both meals led to an increase in TAG storage in the adipose tissue and in the muscle, which became more pronounced, with the increased amount of fat in the meal. On the one hand, glycogen storage was larger in the liver following a high fat meal. On the other hand, the low fat meal elicited an increase in glycogen storage in the muscle.

To conclude, these results confirm that during fasting the internal energy stores were used for energy production. In the fed state, during the analyzed time-course, the internal energy stores were not depleted. Instead meal metabolites were used for energy production. The surplus of energy obtained from the meal was stored in the internal energy stores as glycogen or TAG. As expected, the high fat meal caused an important increase of TAG storage, which was minor after the consumption of a low fat meal, showing the potential detrimental effect of high fat meals consumption in the development of obesity.

### Effect of physical activity at the metabolic level

A regular individual not only eats and fasts during the day, but also does some kind of physical activity, such as slow walking or intense exercising. Similar to the fasting or the fed condition, it is known that during exercise, the metabolism is adapted and that the energy sources used while exercising are dependent on the exercise intensity^22^. Thus, to determine if the model was able to capture key metabolic changes occurring during exercise, exercise was simulated for various intensities and durations. Exercise was mimicked by increasing the ATP consumption in the muscle model. I.e. an intense exercise load of 90% of the maximum oxygen consumption rate (O$_{2,\ \max }$) was simulated by a 42 times fold increase of the ATP consumption, in the muscle, above the basal level. For more details, please refer to the Methods section.

Tables 1 and 2 summarize the effect of exercise on specific fluxes in the adipose tissue, liver and muscle. The change of the fluxes between the different conditions i.e., from a resting state to an exercising state was extracted and compared to literature data. Overall, the model was able to correctly identify flux changes occurring during exercise.

**Table 1:** Effect of exercise on adipose tissue fluxes.

Tissue	Flux	Condition	Literature^29^ 40% O_2, max_	Prediction 40% O_2, max_	Literature^29^ 60% O_2, max_	Prediction 60% O_2, max_
Adipose tissue	Glucose uptake	Exercise	Down	Up	Down	Up
		Post-exercise	Up	Down	Up	Down
	O_2_ uptake	Exercise	Down	Unchanged	Down	Unchanged
		Post-exercise	Up	Unchanged	Up	Unchanged
	Glycerol export	Exercise	Up	Up	Up	Up
		Post-exercise	Down	Down	Down	Down
	Lactate export	Exercise	Up	Up	?	Up
		Post-exercise	Down	Down	?	Down
	Fatty acids export	Exercise	Up	Up	Up	Up
		Post-exercise	Down	Down	Down	Down

Simulation 1: 90 min of 40% O_2, max_ exercise followed by 3 hours resting. Simulation 2: 60 min of 60% O_2, max_ exercise followed by 3 hours resting. The fatty acids flux correspond to the sum of the all fatty acids fluxes in the adipose tissue model. Abbreviations: *Up* flux increase; *Down* flux decrease; *Unchanged* flux unchanged; ? uncertain flux change.

**Table 2:** Effect of steady, and incremental exercise on muscle, and on liver fluxes.

Tissue	Flux	Condition	Literature^30^	Prediction
Liver	Glucose export	Steady exercise	Up	Up
		Resting	Down	Down
		Incremental exercise 70% O_2, max_	Up	Up
		Incremental exercise 80% O_2, max_	Up	Unchanged
		Incremental exercise 90% O_2, max_	Down	Down
	O_2_ uptake	Steady exercise	Up	Up
		Resting	Down	Down
		Incremental exercise 70% O_2, max_	Up	Up
		Incremental exercise 80% O_2, max_	Up	Up
		Incremental exercise 90% O_2, max_	Down	Down
	Lactate uptake	Steady exercise	Up	Up
		Resting	Down	Down
		Incremental exercise 70% O_2, max_	Up	Up
		Incremental exercise 80% O_2, max_	Up	Up
		Incremental exercise 90% O_2, max_	?	Up
Muscle	Glucose uptake	Steady exercise	Up	Up
		Resting	Down	Down
		Incremental exercise 70% O_2, max_	Up	Up
		Incremental exercise 80% O_2, max_	Up	Unchanged
		Incremental exercise 90% O_2, max_	Down	Down
	O_2_ uptake	Steady exercise	Up	Up
		Resting	Down	Down
		Incremental exercise 70% O_2, max_	Up	Up
		Incremental exercise 80% O_2, max_	Up	Up
		Incremental exercise 90% O_2, max_	Up	Up
	Lactate export	Steady exercise	Up	Up
		Resting	Down	Down
		Incremental exercise 70% O_2, max_	Up	Up
		Incremental exercise 80% O_2, max_	Up	Up
		Incremental exercise 90% O_2, max_	Up	Up

Simulation 3: 5 min of 50% O_2, max_ exercise followed by 1h55 of 70% O_2, max_ exercise (steady exercise). After 30 min resting, incremental exercise was simulated. Each phase of the incremental exercise was simulated for 10 min (10 min exercise at 70% O_2, max_, 10 min at 80% O_2, max_, and 10 min at 90% O_2, max_). Abbreviations: *Up* flux increase; *Down* flux decrease; *Unchanged* flux unchanged; ? uncertain flux change.

In the first experiment, exercise performed at 40% O$_{2,\ \max }$ and at 60% O$_{2,\ \max }$ were compared to previous data^29^. The model was able to correctly predict an increase in fatty acids, lactate and glycerol export by the adipose tissue (Table 1). When comparing both simulations (Supplementary Fig. 4), the most intense exercise induced a decrease in glycerol export, and an increase in lactate export and glucose uptake in the adipose tissue. The fatty acids export did not change between the simulations, as its flux was already at the maximum allowed value. The oxygen uptake was not affected by exercise, and was constant throughout both simulations.

In a second experiment, exercise performed in an incremental manner was simulated and the predicted flux changes were compared to previous data^30^. The experimental setup consisted on simulating a steady exercise for 2 h, and after 30 min of recovery the intensity of exercise was incremented from 70% to 90% O$_{2,\ \max }$ every 10 min. Table 2 lists the flux changes occurring for specific fluxes in the liver and in the muscle. The glucose export by the liver increased during incremental exercise until 70% O$_{2,\ \max }$ and at 90% O$_{2,\ \max }$, the glucose export decreased. However, the flux remained larger than the one in the pre-exercise and resting condition. The muscle glucose uptake followed the same pattern as the glucose uptake in liver. The oxygen uptake in the muscle increased proportionally to the exercise intensity. The oxygen uptake in the liver followed a similar pattern, with the exception that at 90% O$_{2,\ \max }$ it decreased. Lactate was secreted in large amounts by the muscle during exercise, and was taken up by the liver. The maximum secretion, and uptake rates were similar at 70% O$_{2,\ \max }$, and at 80% O$_{2,\ \max }$ (only slightly increased). The increase of the exercise intensity to 90% O$_{2,\ \max }$, mainly affected the lactate export in the muscle which increased (Supplementary Fig. 5). All together, the model predictions matched known changes in 90% of the cases.

Supplementary Fig. 6 represents the prediction of the contribution of the different energy sources during incremental exercise. During a fasting resting state, fat derived energy (free fatty acids, and other fat sources: representing the muscle TAG degradation in muscle, and the blood TAG absorption) was the main energy source in the muscle. When exercise started, energy was mainly derived from muscle glycogen degradation, whose degradation rate was proportional to the exercise intensity. On the other hand, the energy supply from fat decreased. At high intensity exercise (90% O$_{2,\ \max }$), the uptake of free fatty acids was completely suppressed, and the muscle glycogen degradation became the main energy source. Fatty acid and glucose oxidation increased during incremental exercise. On the one hand, exercise performed at high intensity led to the decrease of fatty acid oxidation below the resting value. On the other hand, glucose oxidation doubled at high intensity exercise.

In the previous simulations exercise was simulated during a fasting condition. However, nowadays due to the high availability of food, people remain often in a postprandial state during the day. Therefore, we wanted to investigate whether the model was able to show any metabolic changes between exercise performed in the fasting or in the postprandial condition. To this end, 3 simulations were performed: a low fat meal simulation followed by either resting (**M** → **R**) or exercise (**M** → **E**), and exercise followed by a low fat meal (**E** → **M**). Specific tissue fluxes and tissue stores amounts were compared among these three conditions (Supplementary Figs. 7 and 8).

The ingestion of a low fat meal led to an increase in glucose, and TAG uptake in muscle, and to a decrease in fatty acids uptake in muscle, and glycerol secretion in the adipose tissue (Supplementary Fig. 7). In the fed state, the whole-model glucose oxidation increased and the fatty acid one decreased. These effects were apparent in all three simulations after the meal was ingested.

In the **M** → **E** condition, during exercise the glucose uptake, TAG, and fatty acids uptake in the muscle were increased, as well as the glycerol secretion from the adipose tissue. The whole-model fatty acid and glucose oxidation increased more than 5 times, when compared to the **M** → **R** condition. Furthermore, when exercise stopped, the TAG and the fatty acids uptake rate in the muscle decreased but remained above the pre-exercise value.

The model predicted a different effect, if exercise was performed before or after a meal. The muscle glucose uptake was larger in the **M** → **E**, than in the **E** → **M** condition. The adipose tissue secreted larger amounts of glycerol during **M** → **E**, than in the **E** → **M** condition. If exercise was performed before the meal (**E** → **M**), the whole-model fatty acid oxidation was increased and the glucose one was decreased when compared to the **M** → **E** condition.

During exercise, the liver glycogen usage was similar in both conditions. However, in the **E** → **M** condition, the degradation rate of TAG in adipose tissue and glycogen in muscle was larger than in the **M** → **E** condition.

On the other hand, the ingestion of a low fat meal led to a slight storage of energy in the different tissues (Supplementary Fig. 8).

In the next step, a similar analysis was performed to investigate how the ingestion of a high fat meal impacts metabolism during exercise. The results suggest that a high fat diet did not affect dramatically the tissue fluxes (Supplementary Fig. 9). However, a more drastic effect was visible at the tissue energy storage (Supplementary Fig. 10). When compared to the low fat meal, the high fat meal led to an increased storage of TAG and glycogen in the different tissues.

Taken together, these results show the potential of the model to predict the effect of various exercise intensities and the ingestion of meals with different nutrient composition on tissue metabolism.

### Prediction of IEMs specific biomarkers

IEMs are inherited metabolic diseases characterized by an impairment in the metabolic activity of an enzyme or a transporter. If people affected by one IEM have unrestricted diets, toxic metabolites might build up in the body fluids (i.e., blood and urine) and tissues. Therefore, it is important to identify potential biomarkers for all IEMs to get a quicker diagnosis and to avoid health complications at long term^31^. Thus, we simulated the effect of 65 IEMs during different conditions (in the fasting condition and after the ingestion of a low and a high fat meal) and predicted their biomarkers. To validate the results, the set of predicted biomarkers for 17 IEMs was compared to biomarkers previously^32^ manually extracted from OMIM.

**Fig. 4:**
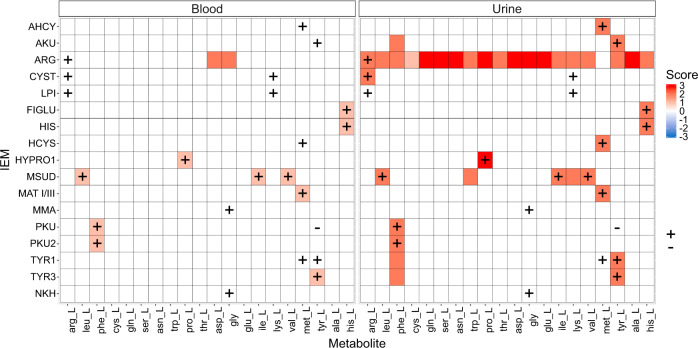
Prediction of amino acids biomarkers in different biofluids for a set of IEMs. In red: metabolites predicted to have increased levels in an IEM. In blue: metabolites predicted to have decreased levels in an IEM. In white: metabolites predicted to remain unchanged in the presence of an IEM. Plus sign (+): metabolite level known to be increased in an IEM. Minus sign (−): metabolite level known to be decreased in an IEM. The data was taken from Shlomi et al.^32^ The score value represents the number of conditions, that the metabolite was predicted to be a biomarker in. As the IEMs were simulated in three different conditions, the absolute score value ranges from 0 to 3. With 0 representing a metabolite not identified as a biomarker, and a score of i.e., 3 representing a metabolite identified as being a biomarker in all 3 simulated conditions (fasting, low fat meal and high fat meal). Abbreviations of IEM names: AHCY = S-adenosylhomocysteine hydrolase; AKU = Alkaptonuria; ARG = Arginase deficiency; CYST = Cystinuria; LPI = Lysinuric Protein Intolerance; FIGLU = Glutamate Formiminotransferase Deficiency; HIS = Histidinemia; HCYS = Homocystinuria; HYPRO1 = Hyperprolinemia Type I; MSUD = Maple Syrup Urine Disease; MAT I/III = Methionine adenosyltransferase I/III deficiency; MMA= Methylmalonic Acidemia (MMA); PKU = Phenylketonuria; PKU2 = Phenylketonuria Type II; TYR1 = Tyrosinemia Type I; TYR3 = Tyrosinemia Type III; NKH = Glycine Encephalopathy/Nonketotic Hyperglycinemia.

Figure 4 shows the predicted biomarkers in the blood and in the urine biofluids for a set of 17 IEMs. At the blood level, the model correctly predicted 10 biomarkers, with only 2 false predictions. The model predictions reached a precision of 83.3%.

We then assumed that biomarkers, generally found in the blood, might also be found in the urine, and we analyzed the results. At the urine level, the model was able to identify known biomarkers, but also other not reported biomarkers. The model predicted a large number of false positive biomarkers in the urine, especially for Arginase deficiency. In the urine, the majority of amino acids, except methionine, were identified as being increased in the Arginase deficiency condition. Thus, we performed a more extensive investigation of this IEM. During the simulation of this IEM, urea synthesis from arginine was knocked-out. Urea synthesis leads to the removal of NH_4_, which is toxic for the human body. In the healthy fasting condition, we observed a basal urea production (Supplementary Fig. 11), due to the protein turnover reaction. After a meal was ingested, the urea production rates increased. This increase did not occur in the unhealthy condition. The knock-out of *Arginase1* gene led to the complete impairment of urea production, in the fasting, but also in the fed condition. Due to this impairment, the amino acids could not be completely degraded and were then excreted through the urine.

To further validate the predictions for Arginase deficiency, known biomarkers were extracted from multiple databases and they were compared against the model predictions (Supplementary Table 1). The model predictions showed good agreement with the literature, if we ignore the fluid where the biomarker was predicted. Ornithine was the only metabolite, which change was not matched with literature at the blood, and at the urine level. Ornithine was predicted to be increased in the blood, and in the urine, however, in literature, it has been reported to be normal, or decreased in the Arginase deficiency condition. This mismatch is explained by the fact that ornithine is also associated with other pathways, i.e., creatine synthesis. In the Arginase deficiency condition, the majority of the amino acids were increased, and the model tried to decrease glycine and arginine levels by converting these amino acids to ornithine and guanidoacetic acid. Guanidoacetic acid was then used to produce creatine which was predicted and also reported to be increased in this condition. Ornithine participates in the synthesis of citrulline, which is involved in the urea cycle. Since the urea cycle was impaired, not all ornithine could be used to produce citrulline. Thus, the system tried to split the accumulation of ornithine by converting some ornithine to citrulline and *γ*-aminobutyric acid and the remaining ornithine was just excreted through the urine or accumulated in the blood. Overall, the model was able to identify more correct biomarkers in the urine, than in the blood. And some known blood biomarkers, were predicted to be biomarkers in the urine instead of the blood i.e., urea, glutamine, while uracil, a known urine biomarker, was predicted to be a biomarker in both fluids. The prediction of some known biomarkers on the urine instead of the blood, and the effect that the Arginase deficiency had on model, is easily explained by the model structure, and its assumptions. The full list of predicted biomarkers for the 65 IEMs, identified in both biofluids, is given in Supplementary Data 2.

The investigation of the other IEMs led to the observation that in 3 out of 5 false predictions, phenylalanine was predicted to be increased, as well as tyrosine.

**Fig. 5:**
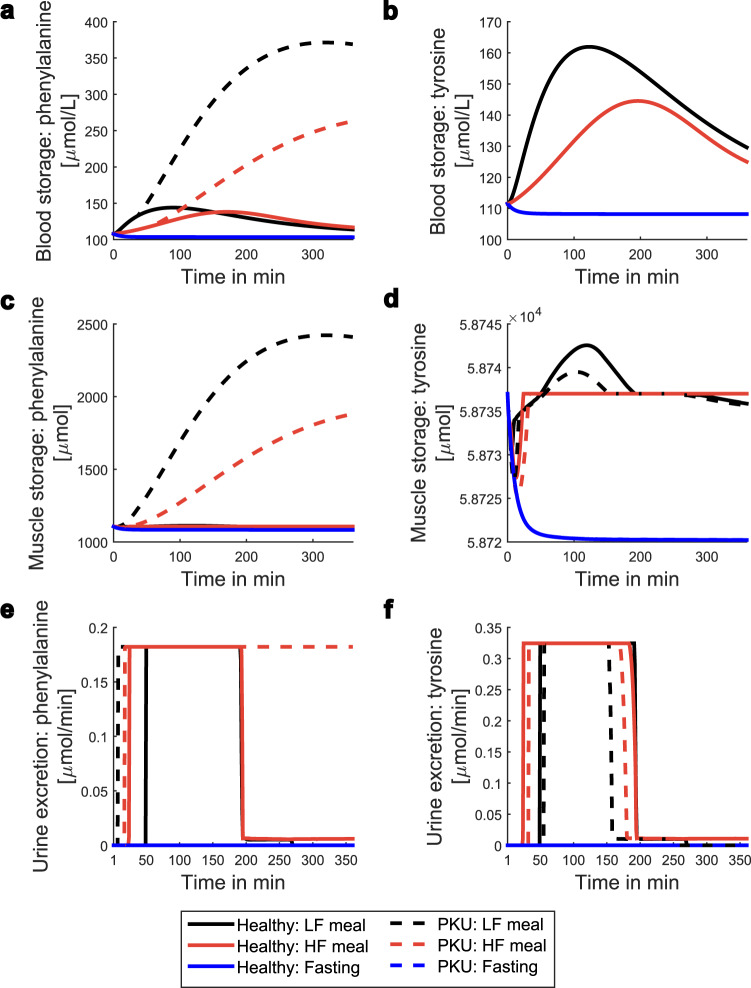
Dynamic profile of phenylalanine and tyrosine during Phenylketonuria and healthy conditions. LF meal = Low fat meal; HF meal = High fat meal; PKU = Phenylketonuria.

Regarding all the false negative results, there were 7 biomarkers that could not be predicted at the blood and at the urine level. Furthermore, we observed that our model predicted less false negative biomarkers when compared to others^32^. However, our approach could not predict tyrosine as a Phenylketonuria (PKU) biomarker. Thus, we investigated the dynamics of tyrosine at the different storage and excretion levels to determine if it decreased in this condition (Fig. 5). Indeed, the tyrosine level at the muscle storage, and the tyrosine excretion rate through the urine were decreased after the ingestion of a meal. However, this decrease was not large enough, and thus this metabolite was not predicted as a biomarker. At the blood level, the tyrosine concentration dynamics was similar between the healthy and the respective PKU condition.

These results show the predictive capacity of this framework, and the importance of simulating and predicting metabolites changes at the intracellular, but also at the extracellular level.

## Discussion

We developed a dynamic multi-tissue model able to reproduce known features of human metabolism. The model was developed in a way, which tried to simplify its structure while still retaining its ability to explain human physiology.

The proposed model was simulated using a dynamic Flux Balance Analysis approach^20^, and optimized for a complex objective function, which relies on three main sub-objectives: (1) maintenance of blood homeostasis. During fasting conditions, blood levels should remain stable, and in the fed conditions they should return to the basal levels within a few hours after the meal. This sub-objective was supplemented by two others: (2) all energy ingested in excess will be stored, i.e., as triacylglycerol in the adipose tissue, and will be used, to a higher extent, when the body goes through periods of food scarcity, i.e., long periods of fasting. Most of the reactions’ flux in constraint-based metabolic models are not constrained, and during the optimization of the model, multiple flux solutions might be equally valid. Thus, a third sub-objective was added, (3) which aim was to prevent sharp changes over time, in the metabolic fluxes. The choice of this multi-objective function allowed the model to mimic quite well the metabolism and the metabolic flow between the three tissues and the blood. Each of the sub-objectives are optimized simultaneously, and can be weighted equally, or not. As an example, in this model, the same weight was selected for each of the blood metabolites. The effect of this assumption led to each metabolite being penalized similarly, as will be further described during the discussion of the IEMs results. In the future, the penalization coefficients could be further tuned to further improve the prediction capacity of the model.

Commonly, reactions flux are constrained with hard bounds. However, due to model incompleteness, experimental errors, and noise, imposing hard constraints on reaction fluxes can lead to infeasible solutions. In order to prevent it, “flexible” constraints^11^ can be applied on the reactions’ bounds. During model optimization, the flux solution will try to remain inside the expected flux range, and it will only deviate from the expected range, if really necessary. Because of the large nature of the model, and the complexity of the objective function, and to avoid infeasible solutions, we decided to apply flexible constraints on the reaction bounds.

One advantage of constraint-based metabolic models is that they necessitate much less parameters, when compared to those models based on ordinary differential equations (ODEs). As described, constraint-based metabolic models rely on the optimization of one objective function, and the application of specific constraints in order to obtain condition specific flux solutions. Due to the small number of parameters, these models can be easily scaled to describe the complete metabolism of one, or more tissues. As demonstrated, the model was able to describe the complete metabolism of three tissues, showing the enormous potential of this approach. The proposed model was validated by simulating different physiological (fasting, starvation, feeding and exercise), and unhealthy conditions (different IEMs).

During the first hours of fasting, the model showed little dynamics, and the metabolic pathways usage was stable. At around 2 days of fasting, the model showed a dynamical switch, when the liver glycogen stores were completely depleted, forcing the system to adapt and choose other energy sources. It is known that liver glycogen depletion occurs after 48 to 60 h of fasting^33,34^, therefore the predicted time of the complete glycogen depletion is in the same range as the reported one. When glycogen stores deplete, the body relies mainly on fatty acids issued from the degradation of adipose tissue TAG^22^, to avoid the depletion of blood glucose. The model was able to capture this metabolic change by predicting an increase in the triacylglycerol synthesis and fatty acid oxidation fluxes. Concomitantly, the glycolysis/gluconeogenesis fluxes decreased, confirming the usage of fatty acids as main energy source.

It has been reported, that the liver is the primary metabolizer of amino acids, with the exception of branched chain ones (leucine, isoleucine, and valine)^22^. However, the aromatic amino acids, phenylalanine, tyrosine, and tryptophan were predicted to be mainly metabolized in the muscle. This was unexpected, as in reality the degradation of these amino acids occurs primary in the liver^22^. This occurred because prior to the model reconstruction, we collected the complete canonical pathway of the aromatic amino acids degradation, and used these reactions as part of the set of core reactions for the contextualization of the tissue models. Thus, this will be addressed in future iterations of this model.

The predicted metabolic pathways activities during the fasting, and the fed condition are in agreement with the literature. It is known that in the fed state, fatty acid oxidation rates decrease^23,24^, and the internal energy stores are no longer used. If energy is ingested in excess, it is stored either as glycogen or TAG^22^. The model predicted that the energy storage pattern after a low, or a high fat meal was different. The results show that a high fat meal elicited a larger energy storage than the low fat meal. This is expected as the high fat meal contained a larger amount of TAG and of glucose.

Furthermore, it has been shown that high levels of blood glucose lead to the increase of the glycolysis rate^25^, which was predicted to be increased in the three tissues. In addition, the increase of the glycolysis rate was proportional to the amount of glucose contained in the meal.

The activity of the ROS detoxification pathway was affected by the fed condition, mostly in the liver and in the muscle, and this effect was more pronounced after the high fat meal. Increased levels of blood glucose, and fatty acids lead to the production of increased amounts of ROS^26,27^. Previously, ROS has been associated with the development of insulin resistance^27,28^, which is the first detectable defect in type 2 diabetic patients and it can occur 10 years before the apparent disease^35^.

During a normal day, individuals go from a state of fasting to feeding, and also do some kind of physical activity, which can be done either in the fasting state or after eating. Therefore, to further validate the model, we predicted and partially validated the effect of the exercise intensity and the meal composition on metabolism. This was achieved by modifying the ATP consumption in the muscle, to reflect the exercise intensities of interest. First, we determined by how much the ATP consumption in the muscle would need to be increased to reach 90% of the maximal O_2_ uptake. All the other exercise intensities were calculated linearly. The approach used to calculate the different exercise intensities relies on two main assumptions. The first assumption is that an average individual was used in this work. In the future, the model could be further adapted to represent the metabolism of athletes, by i.e., adapting the maximal O_2_ uptake. The second assumption, which was used due to the lack of data in this field, assumes that the linear increase of ATP consumption in the muscle, leads to the linear increase of O_2_ uptake in humans.

It has been previously established that fat, and carbohydrates are the main energy sources during exercise, and depending on the intensity of the exercise, one source is favored over the other. While at lower to moderate exercise intensities, fatty acids are the main source^36,37^, at high intensity glycogen becomes the main one^38^. The developed model was able to correctly reflect this switch in energy source depending on the intensity level. It correctly predicted that the muscle glycogen usage was proportional to the exercise intensity^38^. The fatty acids uptake in the muscle reached the maximum flux at 60% O$_{2,\ \max }$, and remained at this value for moderate to high intensity exercise. At 90% O$_{2,\ \max }$, fatty acids uptake was completely suppressed. These results suggest that the maximum flux rate used was too small and increasing the value of this flux should be foreseen for future simulations.

Furthermore, at the highest exercise intensity, fatty acids were no longer taken up, and the internal and the external TAG usage was decreased, together with the fatty acid oxidation rate, which decreased below the basal value. It has been reported that the maximal fat oxidation rate lies between 40.8% and 75% of the O$_{2,\ \max }$^39,40^, which is close to the maximal predicted fatty acid oxidation rate (around 80% O$_{2,\ \max }$). In addition, at this intensity, glycogen became the main energy source, as previously reported by van Loon et al.^38^, and the glucose oxidation rate doubled.

Performing exercise in the fasting state has been suggested to be more favorable regarding the lipid metabolism^23^. The proposed model correctly predicted that fatty acid oxidation rate was larger if exercise was performed before a meal (**E** → **M**), rather then after a meal (**M** → **E**)^23^. We demonstrated that performing exercise during fasting promotes fatty acid oxidation, through the increase the TAG degradation rates. If a meal was ingested after the exercise, the TAG level in the adipose tissue remained lower when compared to the level where a meal was ingested prior to the exercise. This effect was similar for both meals. Carbohydrates ingestion after exercise allows the glycogen stores to replenish^41^. In the model, this effect was more pronounced when a high fat meal was ingested before or after exercising. It is important to note again that, not only this meal contained more TAG, but also more glucose.

Overall, these results suggest that more TAG is burned in the adipose tissue, if exercise is performed in the fasting state. If a meal is ingested after exercising, it allows the slight replenishment of glycogen, and simultaneously, it keeps the adipose tissue TAG levels below the **M** → **E** level.

These results can be relevant for the treatment of human obesity. To maximize fat oxidation and potentially fat loss, exercise should be performed in the fasting state, and at low to medium intensity levels, as for high intensities, fatty acid oxidation was predicted to be almost suppressed.

After analyzing the metabolic effect of different physiological conditions, the model was further validated, by predicting biomarkers for a set of IEMs and by comparing them to literature data. The model mainly predicted urine biomarkers, which is explained by the blood homeostasis assumption used to constrain the model. Metabolites could be exported through the urine, if the exporting rate was kept within the healthy range, while their storage in the blood was penalized. Therefore, it was preferable to export biomarkers through the urine, instead of accumulating them in the blood.

The model predicted blood biomarkers, that were also predicted as urine biomarkers. This happens when the urine secretion rate is at the maximum allowed flux, or outside the healthy range. When the urine secretion fluxes are saturated, the model minimizes the objective function value by splitting the penalization among different reactions. Thus, the metabolites that accumulate in the blood are also exported through the urine. To summarize, the model only predicted a blood biomarker, if the respective urine metabolite was also predicted as a potential biomarker.

Arginase deficiency led to the prediction of almost all urine amino acids as potential biomarkers. During this condition, urea synthesis was completely impaired. The urea synthesis pathway is involved in the detoxification of NH_4_, which is one by-product produced during the degradation of amino acids. Due to the impairment on urea synthesis, NH_4_ could not be removed through the urea synthesis pathway. Thus, the model tries to remove NH_4_ from the system by eliminating it through the urine. When this elimination is saturated, NH_4_ might accumulate in the blood. As the final step of the amino acids degradation is impaired, amino acids are eliminated through the urine, and to some extent they are stored as protein in each tissue, even if protein storage is penalized. One way to avoid this effect would be to have a specific penalization coefficient for each metabolite, which would reflect the preference for the elimination, or the accumulation of the metabolite. However, this kind of data would be very hard to obtain. E.g., the biomarkers data, collected in Supplementary Table 1, show that the biomarker change is quite homogeneous among the databases, but not all biomarkers could be retrieved in all the databases. Some biomarkers are known to be present in the blood, and in the urine, while other biomarkers could only be retrieved in the urine i.e., uracil, or orotic acid. These results suggest that either these metabolites have not still been identified as blood biomarkers, or they are preferentially secreted through the urine.

The model predicted 5 false positive biomarkers at the urine level, of which 3 corresponded to the increase of phenylalanine in 3 IEMs (Alkaptonuria, Tyrosinemia Type I, and Type III). In these conditions, the tyrosine degradation pathway is impaired and this impairment leads to the known accumulation of tyrosine. Phenylalanine, which is the tyrosine precursor, will also accumulate as tyrosine cannot be degraded, and will be secreted through the urine to avoid any blood concentration changes. Similar to the Arginase deficiency, this occurs because the model penalizes big changes at the blood level, and favors the sum of small changes. Thus, the objective function value is lower if the model accumulates small amounts of the two amino acids, instead of having a high accumulation of tyrosine.

Two other false positive biomarkers were predicted for Maple Syrup Urine disease (MSUD), and corresponded to lysine and tryptophan. After an in depth investigation, we observed that the *DLD* gene associated with MSUD, also regulates one reaction belonging to the downstream degradation pathway of lysine. This impairment in the lysine degradation pathway leads to the possible accumulation of oxoadipic acid, which is also the degradation product of tryptophan. Thus, an increase in the levels of lysine and tryptophan was not surprising, because of the impairment in the degradation pathways of these two amino acids. These results might suggest that either these amino acids have not yet been reported as potential biomarkers, or that the degradation pathway of these two amino acids is less affected than the one from the branched chain amino acids, potentially because an unknown enzyme might be able to compensate for this loss.

We have demonstrated that this approach shows good agreement with literature data and allows to prioritize potential biomarkers when performing metabolomics analysis.

In conclusion, we developed a dynamic multi-tissue model which not only captures the physiological effects, but is also able to identify potential biomarkers. This framework can be applied in the field of personalized sports nutrition, such as in the improvement of sports performance. This could be done by predicting which diet would elicit the faster muscular glycogen and protein storage repletion. In addition, this approach can be extended to the analysis and biomarker prediction of other metabolic diseases, by performing gene knock-outs for each gene present in the model. In addition, personalized models of any metabolic disease could be contextualized with individual expression data and metabolomics data, which would serve as a basis for the development of personalized treatments and adapted meals for each type of studied disease.

## Methods

While genome-scale reconstructions of human metabolism are readily available^1–7^, high quality models of individual tissues are still rare. However, algorithms for automatic reconstruction of draft context-specific models are available and can be used to create a starting point for tissue specific models, with the FASTCORE family of algorithms having shown the ability to generate good quality draft models^42^. Here, an outline about how these algorithms were adapted to generate draft models for three tissues (liver, muscle and adipose tissue) is presented. Further details are given about how the quality of the generated models was assessed. Finally, the approach used to connect these models and the concepts applied to interrogate the system are described.

### Data collection and processing

Tissue specific transcriptomics data from the GEO database was extracted for the three tissues of interest. Tissue datasets were selected by filtering for the following keywords: liver, skeletal muscle, and subcutaneous adipose tissue. Only transcriptomics datasets in the healthy condition, generated by the platform U133 Plus 2.0, were selected (Supplementary Table 2). This platform was selected, as Barcode was compatible to it. The data was processed using the Gene Expression Barcode^43–45^ discretization approach, and integrated in the FASTCORMICS workflow^46^. In short, the microarrays were read into R version 3.2.0, with the affy package (1.48.0). They were subsequently normalized with the fRMA package (1.22.0), and then discretized with Barcode using the hgu133plus2frmavecs vector (1.5.0).

### Tissue models’ reconstruction

Three healthy tissue models (liver, skeletal muscle, and subcutaneous adipose tissue) were reconstructed from Recon2.04^3^. Prior to the reconstruction, Recon2.04 was mass and charge balanced, and some reactions were corrected. All the modifications performed are listed in Supplementary Data 3. The tissue-specific models were reconstructed using a modified FASTCORMICS workflow^46^. In addition to the data derived from the gene expression profiles, reactions from canonical pathways known to be active in the tissues and additional transporters were added as core reactions. They were added to avoid gaps in the canonical pathways and to make sure that known transporters were present in the final model reconstruction, since FASTCORMICS tends to remove as many unsupported transport reactions as possible.

These supplementary core reactions were comprised of:

- Reactions belonging to canonical pathways which should be present in the tissue models i.e., degradation of amino acids.
- Reactions not expressed based on the transcriptomic level, but expressed based on proteomics data (data from the Human Protein Atlas database).
- Reactions involved in the recycling of co-factors.

The full list of supplementary reactions is given in Supplementary Data 4.

### Tissue models’ evaluation

Each reconstructed model was evaluated by testing tissue-specific functions based on the physiological functions previously published by Gille et al.^18^ (see Supplementary Data 1). The reconstruction and the model function testing was performed iteratively. Meaning that, if a function test was not successful, gaps were searched in the reconstructed tissue model and filled in order to allow the function to be carried out.

### Literature data

A list of human healthy tissue uptake, and release fluxes for different metabolites was collected from literature. In addition, data from other mammals (e.g., mice for liver amino acids exchange fluxes) was used, if human data was not available. All included fluxes and their references are listed in Supplementary Data 5. All fluxes are given in *μ*mol per min.

### Coupling the three tissue models

Tissue coupling is important to allow a systematic investigation of the reconstructed model, which was achieved by the following approach:

In the first step, after each tissue model was reconstructed, transport reactions between the cytosol and the extracellular space were added for metabolites which were present in the model cytosol and have already been detected in either blood or urine, according to the data from the human metabolome database (HMDB)^47–50^. For each of these metabolites, Recon2.04^3^, and Recon3^5^ were searched for possible transport reactions. If the reaction was present, then it was added to the model, if not the following transport reaction was added: *met*[*c*] ↔ *met*[*e*]. These transporters were added because during the reconstruction process many transporters were removed, and some IEMs biomarkers (either in urine or in blood) were metabolites which were only present in the cytosol of the models.

In the second step, a blood compartment was added to allow metabolite exchange between the different tissue models. Furthermore, a blood pool, and a virtual gut, mimicking food absorption (Fig. 1), from where nutrients could be absorbed to the blood, were added. Mean blood and urine concentrations in healthy, and unhealthy adults were collected from HMDB^47–50^, together with the respective ranges.

The blood concentrations were then converted to amounts, assuming a total blood volume of 5 L (Supplementary Data 6).

In the third step, the urine concentrations were converted to fluxes. The conversion was performed as follows:

- The metabolite concentrations in urine were given in μmol per mmol of creatinine
- The healthy creatinine amount secreted over a period of 24 h in urine was gathered^51^ (9.4 ± 2.68 mmol per 24 h)
- The 24 h creatinine value was converted from mmol per 24 h to mmol per min
- The urine metabolite concentrations were multiplied by the creatinine amount to obtain metabolite secretion fluxes (Supplementary Data 7)

Finally, glycogen stores were added to the liver, and the muscle models, while triacylglycerol (TAG), and protein stores were added to the adipose tissue, liver, and the muscle models. Tissue weights, and tissue stores initial conditions are listed in Supplementary Table 3, and Supplementary Table 4, respectively. The tissue weights employed correspond to a healthy 30-year-old Caucasian European male, weighing 70 kg, and measuring 176 cm, and the initial conditions correspond to usual values found in healthy non fasting men. An ATP demand and a protein turnover reaction (Supplementary Tables 5 and 6, respectively) were added to each tissue model, and were based on the biomass reactions from Bordbar et al.^8^. The protein production reaction was determined as follows: the average of the protein turnover reactions among the three tissues was calculated. The sum of the amino acids coefficients was scaled to 1 and the protein production was made ATP consumption dependent. In addition to the protein stores, amino acids stores were added to the muscle model (Supplementary Table 7). All exchange reactions were charge balanced to avoid losing or gaining charges in the system, and a buffering reaction was added to the blood compartment (H[bl] + HCO_3_[bl] ↔ H_2_O[bl] + CO_2_[bl]). A general overview of the model structure can be found in Fig. 1.

### Overview of the objective function

A common issue for metabolic modeling in mature higher organisms is the selection of an appropriate objective function that properly reflects the aim of metabolism. The modeling approach proposed here was based on three main assumptions:

1. That metabolism aims to maintain a blood concentration homeostasis.
2. That energy might be stored if in excess, to be used during scarcity conditions.
3. That metabolic changes are smooth and not sharp.

This objective must then be subjected to limitations and requirements, such as flux bounds or known turnover and maintenance costs. Extensive literature search was performed to collect tissue specific metabolic fluxes to constrain the multi-tissue model (Supplementary Data 5). All the fluxes were converted to total tissue flux per minute, using the values listed in Supplementary Table 3. Incorporation of these constraints can be achieved either by fixing hard bounds, or by applying penalty terms on the model objective based on violations of these terms, as suggested by Hyötyläinen et al.^11^.

To fulfill the assumed objective, a two step approach was used. In the first step, the model is optimized to achieve homeostasis, and to store energy sources while already aiming for some smoothness. In the second step, the remaining reactions are optimized to achieve a smooth transition.

### First optimization step: objective definition

1. The main objective (blood metabolite homeostasis, see Supplementary Fig. 12) requires a blood metabolite pool to remain as constant as possible over time. This is achieved as follows: first, given a basal blood metabolite level Amount^*m*, basal^ and a blood metabolite level Amount^*m*, t^, in time step *t*, then the deviation of the blood metabolite level from its basal level in time step *t* is Amount^*m*, dev, *t*^ = Amount^*m*, *t*^ − Amount^*m*, basal^. Further, it is assumed that reaction *Ex*_*m*_, with associated flux ${v}_{{\mathrm{Ex}}}^{m}$, is the exchange reaction between the blood storage, and the blood compartment. Then, the following constraints can be added to the model:
  1 $$\begin{array}{*{20}{l}}{{\mathrm{Amount}}}^{m,\ {\mathrm{dev}},\ t}&\le &{v}_{{\mathrm{Ex}}}^{m}\cdot {{\mathrm{TimeStep}}}_{t}-{a}_{{\mathrm{violation}}}^{m,\,{\mathrm{basal}}}&\le &{{\mathrm{Amount}}}^{m,\ {\mathrm{dev}},\ t}\\\\ -\infty &\le &{a}_{{\mathrm{violation}}}^{m,\,{\mathrm{basal}}}&\le & +\infty \end{array}$$
  where TimeStep corresponds to a time step of 1 min. This time step was selected according to Krauss et al.^52^ and Wadehn et al.^9^ and was used for all the performed simulations. By adding the square of ${a}_{{\mathrm{violation}}}^{m, \ {\mathrm{basal}}}$, to the quadratic objective (i.e., minimizing the square), the system aims to minimize the deviation of blood metabolites from their basal levels.
2. Another aim of the model is to replenish transient stores of the organism (Supplementary Fig. 13), such as the glycogen and fat stores in the individual tissues. In addition, the degradation of protein in the different tissues can be used to accommodate protein turnover. During a meal, amino acids might be stored as protein, but neither the protein storage, or degradation is preferential. These stores are added as a linear or quadratic part of the objective as:
  2 $$\begin{array}{lcc} \mathop {\sum}\limits_{{\mathrm{store}}\in C,\,P}{\beta }_{{\mathrm{store}}}\cdot {{v}_{{\mathrm{degradation}}}^{{\mathrm{store}}}}^{2}+ \mathop {\sum}\limits_{{\mathrm{store}}\in C}{\alpha }_{{\mathrm{store}}}\cdot {v}_{{\mathrm{replenish}}}^{{\mathrm{store}}}+ \mathop {\sum}\limits _{{\mathrm{store}}\in P}{\alpha }_{{\mathrm{store}}}\cdot {{v}_{{\mathrm{replenish}}}^{{\mathrm{store}}}}^{2}\end{array}$$
  with the respective *α* and *β* values (please refer to Supplementary Table 8) selected in such a way that conversion from one store to another is not beneficial to the objective, and that glycogen replenishment is preferential to fat production.
3. This objective is supplemented by an aim for smoothness which is defined as:
  3 $$\begin{array}{*{20}{l}}{{\mathrm{Flux}}}^{a,\ t-1}({v}^{a})&\le &{v}^{a}-{v}_{{\mathrm{violation}}}^{a}&\le &{{\mathrm{Flux}}}^{a,\ t-1}({v}^{a})\\ \\ -\infty &\le &{v}_{{\mathrm{violation}}}^{a}&\le & + \infty \end{array}$$
  where *t* − 1 corresponds to the previous time step, and *a* represents each reaction in the model.

### First optimization step: flexible constraints

In addition to the aims detailed above, there are several flexible constraints (Supplementary Fig. 14), which are implemented such that they also influence the objective value. These constraints are:

1. Constraints on the fluxes of reactions with known flux ranges (i.e., all reactions from *R*_*K*_), which are defined as:
  4 $$\begin{array}{*{20}{l}}{{\mathrm{Flux}}}_{{\mathrm{lit}}}^{i,\,{\mathrm{min}}}({v}^{i})&\le &{v}^{i}-{v}_{{\mathrm{violation}}}^{i}&\le &{{\mathrm{Flux}}}_{{\mathrm{lit}}}^{i,\,{\mathrm{max}}}({v}^{i})\\ \\ -\infty &\le &{v}_{{\mathrm{violation}}}^{i}&\le & + \infty \end{array}$$
  where ${{\mathrm{Flux}}}_{{\mathrm{lit}}}^{i,\,{\mathrm{min}}}$ and ${{\mathrm{Flux}}}_{{\mathrm{lit}}}^{i,\,{\mathrm{max}}}$ are the minimal and maximal healthy literature values for flux *i* from reactions in *R*_*K*_, respectively. ${v}_{{\mathrm{violation}}}^{i}$ can then be used as a representation of the violation of the bounds, and its square is added to the objective as a penalty term. These constraints are introduced for all *i* ∈ *R*_*K*_. For all supplementary transport fluxes (*R*_CS_), and protein degradation reactions (*R*_PD_), similar constraints with ${{\mathrm{Flux}}}_{{\mathrm{lit}}}^{i,\,{\mathrm{min}}}={{\mathrm{Flux}}}_{{\mathrm{lit}}}^{i,\,{\mathrm{max}}}=0$ were employed.
2. Known metabolite levels are also incorporated by a similar mechanism. Metabolite amounts are converted into fluxes based on their amount range:
  5 $$\begin{array}{*{20}{l}}{{\mathrm{Amount}}}_{{\mathrm{lit}}}^{m,\,{\mathrm{min}}}-{{\mathrm{Amount}}}^{m,\ t}&\le &{v}_{{\mathrm{Ex}}}^{m}\cdot {\mathrm{TimeSte{p}}}_{t}-{a}_{{\mathrm{violation}}}^{m}&\le &{{\mathrm{Amount}}}_{{\mathrm{lit}}}^{m,\,{\mathrm{max}}}-{\mathrm{{Amount}}}^{m,\ t} \\ \\ -\infty &\le &{a}_{{\mathrm{violation}}}^{m}&\le &+\infty \end{array}$$
  where Amount^*m*, *t*^ correspond to the current metabolite amount at time *t*. These constraints are constructed for all metabolites *m* from *B* and *M*_AA_.
  The square of the violation of known ranges ${a}_{{\mathrm{violation}}}^{m}$ is then added to the objective.
3. Modified quadratic coefficients for specific reactions (Supplementary Table 9)
  Coefficients were modified according to the function that the reaction should fulfill. (1) The maximum allowed flux for the liver glycogen storage reaction was very high, as this reaction can carry a higher or a lower flux depending on the physiological state of the body. Therefore, a flexible constraint, based on the fasting rates, was set to a very high coefficient value to further penalize the deviation from these bounds. The same procedure was employed for the CS reactions. (2) The flexible bounds for the oxygen uptake in the muscle are bounds in the resting state. However, during the simulation of exercise, the model needs to uptake more oxygen. Thus, the uptake of oxygen was slightly penalized. (3) When going from one metabolic state to another, the model needs to adapt its metabolism. Since it might be impossible for the model to have a smooth transition from the previous to the current time step, a coefficient of 0.001 was given to still penalize the deviation from the previous time step, while still allowing some flexibility during the metabolic adaptation. (4) All the other coefficients for all the other reactions included in the quadratic objective function were set to a value of 1.

This leads to the following objective function:

6 $$\begin{array}{*{20}{l}}Z\,=\,\mathop {\sum}\limits_{m\in B,\,{M}_{{\mathrm{AA}}}}{{a}_{{\mathrm{violation}}}^{m,\ {\mathrm{basal}}}}^{2}\quad +\quad \mathop {\sum}\limits_{\begin{array}{c}a\in {R}_{K},\ {R}_{B},\ {R}_{{\mathrm{CS}}},\ {R}_{C},\end{array} {R}_{{\mathrm{MAA}}},\ {R}_{{\mathrm{PS}}},\ {R}_{{\mathrm{PD}}}}\tau \cdot {{v}_{{\mathrm{violation}}}^{a}}^{2}\quad +\quad \mathop {\sum}\limits_{i\in {R}_{K},\,{R}_{{\mathrm{PD}}},\,{R}_{{\mathrm{CS}}}}\tau \cdot {{v}_{{\mathrm{violation}}}^{i}}^{2}\quad +\quad \mathop {\sum}\limits_{m\in B}{{a}_{{\mathrm{violation}}}^{m}}^{2}\\\quad+\,\mathop {\sum}\limits_{{\mathrm{store}}\in C,\,P}{\beta }_{{\mathrm{store}}}\cdot {{v}_{{\mathrm{degradation}}}^{{\mathrm{store}}}}^{2}\quad +\quad \mathop {\sum}\limits _{{\mathrm{store}}\in C}{\alpha }_{{\mathrm{store}}}\cdot {v}_{{\mathrm{replenish}}}^{{\mathrm{store}}}\quad +\quad \mathop {\sum}\limits_{{\mathrm{store}}\in P}{\alpha }_{{\mathrm{store}}}\cdot {{v}_{{\mathrm{replenish}}}^{{\mathrm{store}}}}^{2}\end{array}$$

With *B*, *C*, *P*, *R*_*K*_, *R*_CS_, *R*_*PS*_, *R*_PD_, *R*_*B*_, *R*_MAA_, *R*_*C*_ as defined in Table 3. Because of the objective being a minimization, all elements in the quadratic terms will assume an absolute minimal value. Overall, the objective simultaneously minimizes the deviation from the steady state condition, while replenishing the stores. The choice for the individual *α*_store_ and *β*_store_ values, detailed in Supplementary Table 8, ensures that it is not favorable to convert one store to another.

**Table 3:** List of the abbreviations used to define the constraints during the optimisation step.

Abbreviation	Definition
*R*	All reactions present in the model
*R*_*K*_	The set of reactions with known ranges, such as: tissue exchangers, urine secretion reactions
*B*	The set of metabolites with known basal concentration levels or ranges in the blood
*R*_*B*_	The set of blood storage reactions associated with the metabolites in *B*
*M*_*AA*_	The set of metabolites in the muscle amino acid storage
*R*_*MAA*_	The set of muscle amino acids storage reactions associated with metabolites in *M*_*AA*_
*R*_*CS*_	The set of added transport reactions from the cytoplasm to the extracellular space (e.g. met[c] ↔ met[e]) The protein sources which are stored and degraded
*P*	The set of protein sources which are stored and degraded
*R*_*PD*_	The set of protein degradation reactions
*R*_*PS*_	The set of protein storage reactions
*R*_*NU*_	The set of nutrient uptake reactions (→ glc[bl])
*C*	The set of carbon sources which are stored and degraded
*R*_*C*_	The set of reactions associated with storages in *C*

### First optimization step: hard constraints

In addition to the flexible constraints, which are represented as penalty terms in the objective function, the following constraints are added to the problem:

1. Internal steady state:
  7 $$S\cdot v=0$$
2. Maximal oxygen uptake rate:
  8 $${{\mathrm{maxUptake}}}_{{{\mathrm{O}}}_{2}}\le {v}_{{\mathrm{E{X}}}_{{{\mathrm{O}}}_{2}}}\le 0$$
3. Maximal CO_2_ secretion rate:
  9 $$0\le {v}_{{\mathrm{E{X}}}_{{\mathrm{C{O}}}_{2}}}\le {\mathrm{maxExpor{t}}}_{{\mathrm{C{O}}}_{2}}$$
4. Maximal protein degradation rate:
  The maximal protein degradation rate was split over all tissues, with the flux sum of all tissues being limited by a maximal amount.
  10 $${v}_{{\mathrm{degradation}}}^{{\mathrm{muscle}}}+{v}_{{\mathrm{degradation}}}^{{\mathrm{hep}}}+{v}_{{\mathrm{degradation}}}^{{\mathrm{fat}}}\le {v}_{{\mathrm{degradation}},\ {\mathrm{max}}}$$
  The same was done for the maximal CO_2_ secretion rate, and the maximal transport of fatty acids (Supplementary Data 5).
5. Maintenance energy demands for ATP, and protein turnover in each tissue.
  Maintenance energy demands are represented by enforced fluxes for ATPase reactions: ATP + H_2_O → H^+^ + ADP + Pi
  11 $$\begin{array}{lll}{\mathrm{ATPas{e}}}_{{\mathrm{Hep}}}\,=\,{\mathrm{He{p}}}_{{\mathrm{maintenance}}}\\ {\mathrm{ATPas{e}}}_{{\mathrm{Muscle}}}\,=\,{\mathrm{Muscl{e}}}_{{\mathrm{maintenance}}}\\ {\mathrm{ATPas{e}}}_{{\mathrm{AdiposeTissue}}}\,=\,{\mathrm{AdiposeTissu{e}}}_{{\mathrm{maintenance}}}\end{array}$$
6. Maximal urine secretion rate:
  12 $$0\le {v}_{{\mathrm{E{X}}}_{U}}\le {\mathrm{maxExpor{t}}}_{U}$$
  where maxExport_*U*_ represents the maximum secretion rate among the healthy, and the diseased conditions.
7. Constraints on the maximal/minimal available amounts for blood metabolites, and muscle amino acids:
  The minimal and the maximal available amounts, from all storages (blood, or amino acid tissue storages) are incorporated in two different ways. For all blood metabolites which are available in the food (NU), the uptake rates (through reactions of the form: *R*_*f*_: met_f_[blood] ↔ ) are restricted by:
  13 $${v}^{f}\le -{v}_{{\mathrm{max}}}^{f}$$
  with ${v}_{{\mathrm{max}}}^{f}$, representing *v*_*m*_ as defined in Eq. (22). The solution became infeasible when a meal was applied, after the model had been simulated in the fasting or exercising condition. Thus, to avoid the infeasibility of the flux solution, Eq. (13) was modified in the following way:
  $${\mathrm{min}}(-{v}_{{\mathrm{max}}}^{f},{v}^{f,\ t-1})\le -{v}_{{\mathrm{max}}}^{f}$$
  where the minimal value between the flux given by Eq. (22) and the previous flux value *v*^*f*, *t*−1^ is used as lower bound for the exchange reaction of metabolites present in food.
  This constraint is used, instead of constraint Eq. (13) during the following conditions: during the first 8 min, if a low fat meal is applied after the simulation of fasting, and during the first 24 min if it is applied after the simulation of exercise. Furthermore, this constraint is applied during the first 15 min, if a high fat meal is applied after the simulation of fasting and during the first 44 min, if it is applied after the simulation of exercise. This time was the time necessary for the model to adapt its metabolism between conditions.
  For all other blood metabolites *j*, the following constraints are used:
  14 $$-\frac{{j}_{{\mathrm{storage}}}}{{\mathrm{TimeStep}}}\le {v}^{j}$$
  with *j*_storage_ representing the current amount of the metabolite stored in the respective storage, and *v*^*j*^ is the flux through the respective exchange reaction.
  For all amino acids in the muscle stores *j*, the following constraints are used:
  15 $$-\frac{{j}_{{\mathrm{storage}}}}{{\mathrm{TimeStep}}}\le {v}^{j}\le {v}_{{\mathrm{max}}}^{j}-\frac{{j}_{{\mathrm{storage}}}}{{\mathrm{TimeStep}}}$$
  with *j*_storage_ representing the current amount of the metabolite stored in the respective storage, *v*^*j*^ is the flux through the respective exchange reaction, and ${v}_{{\mathrm{max}}}^{j}$ is the maximum allowed amount of amino acid storage.

Overall the following quadratic problem will be solved:

16 $$\begin{array}{ll}{\mathrm{min}}\,{\mathrm{Z}}&\\ s.t.\,:&\\ &{\mathrm{Constraints}}\,(7)\,to\,(15)\end{array}$$

### Second optimization step

Since the optimal flux in the first optimization step is not necessarily unique, and a biological model commonly has rather smooth transitions, a secondary objective is employed. For time zero, as the original flux distribution is not available, a quadratic flux minimization is performed as its solution is known to be unique^53,54^.

17 $${\mathrm{min}}\sum _{i\in R}{{v}^{i}}^{2}$$

For all later time points, the model aims to achieve a solution as close to the previous as possible, to allow a smooth transition. The use of quadratic constraints was avoided, as they require specific properties to be solvable, and these properties are not guaranteed. In this step, the original objective value is not used to constrain the problem. Instead, constraints are put on the individual components of the objective, to ensure that the objective value from the objective function (Eq. (6)) is retained. Therefore, only the reactions which were not part of it are optimized:

18 $${\mathrm{min}}\sum \tau \cdot {({v}_{uc}-{v}_{uc}^{{t}\,-\,{1}})}^{2}\quad uc\,\notin\, {R}_{K},\,{R}_{B},\,{R}_{{\mathrm{MAA}}},\,{R}_{{\mathrm{CS}}},\,{R}_{{\mathrm{PD}}},\,{R}_{{\mathrm{PS}}},\,{R}_{C}$$

Subjected to:

19 $${v}_{j}={v}_{j}^{{\mathrm{Opt}}1}\quad \,\,j\in {R}_{K},\,{R}_{B},\,{R}_{{\mathrm{MAA}}},\,{R}_{{\mathrm{CS}}},\,{R}_{{\mathrm{PD}}},\,{R}_{{\mathrm{PS}}},\,{R}_{C}$$

### Addressing numerical difficulties during the optimization steps

A set of Cplex parameters were modified to address the numerical difficulties occurring during the optimization steps.

- Cplex.Param.timelimit.Cur = 60 ⋅ 10
- Cplex.Param.barrier.algorithm.Cur = 1
- Cplex.Param.barrier.convergetol.Cur = qp.Param.barrier.convergetol.Cur ⋅ 10
- Cplex.Param.emphasis.numerical.Cur = 1

In addition, if the solver was still unable to identify an optimal solution, the presolver was turned off, which in some cases helped to optimize the model.

- Cplex.Param.preprocessing.presolve.Cur = 0

Furthermore, because of the numerical difficulties, occasionally the solution became infeasible during the second optimization step (flux minimization), while it was optimal during the first optimization step. Therefore, for each time step the solution from the first optimization step was used to update all the stores, i.e., the blood concentrations and the internal tissue stores.

### Simulating the model

The multi-tissue model was simulated using a dynamic Flux Balance Analysis-based approach^20^, and optimized for the objective function described above, using the COBRA Toolbox for MATLAB and the ILOG Cplex solver. Each simulation was performed for a period of time, i.e., 360 min, using a time step of 1 min.

The pipeline for the simulation of each time step is described by the following steps:

1. Initialize the model (only for time = 1 min)
2. Calculate the food amount that is absorbed from the gut to the blood using Eq. (21)
3. Update the blood amounts
4. Calculate *v*_*m*_ using Eq. (22)
5. Set the new flux constraints
6. Perform the first optimization step
7. Perform the second optimization step
8. Update all the metabolite amounts in the blood and in the tissues (i.e., internal energy stores, blood metabolites, amino acids in the muscle, etc.) using the flux solution from step 6
9. Restart from step 2 until the end of the simulation, i.e., time = 360 min

### Simulating meals with different fat contents

Diet information, and blood metabolite profiles were obtained from Milan et al.^21^. In this dataset, 20 metabolite profiles (18 amino-acids, glucose and TAG) were available. These metabolites were measured at baseline, and then every 60 min for 300 min, after ingestion of a low or a high fat meal. The average metabolite profiles of young individuals were used to fit the model.

The foods included in the low and in the high fat meal were converted to nutrients using the Frida database^55^. If they could not be found in this database, the SelfNutrition database^56^ was used instead.

If starch values were present in the database, it was assumed that half of the starch was starch1 and the other half was starch2, to match the starch metabolites in Recon2.04. If the starch value was not available from the database, then the following calculation was performed:

20 $${\mathrm{Total}}\,{\mathrm{starch}}={\mathrm{Total}}\,{\mathrm{carbohydrates}}-{\mathrm{Total}}\,{\mathrm{sugars}}-{\mathrm{Dietary}}\,{\mathrm{fiber}}$$

This value was assumed to be the value of starch missing. Similarly, it was assumed that half of this value corresponded to starch1 and the other half to starch2.

Starch1, starch2, disaccharides, fructose, and galactose were converted to glucose with the conversion factor of 11, 3, 2, 1, and 1, respectively. The conversion step was performed as the carbohydrate hydrolysis in the gut was not explicitly modeled.

Previously, Hovorka et al.^19^ employed Eq. (21) to describe the glucose absorption in the gut. In this work, Eq. (21) was discretized, and employed to describe all the nutrients absorption from the gut to the blood.

Hovorka’s equation:

21 $${U}_{m}(t)=\frac{{D}_{m}\cdot {A}_{m}\cdot t\cdot {e}^{\frac{-t}{{t}_{max}^{m}}}}{{({t}_{max}^{m})}^{2}}$$

where *U*_*m*_(*t*) is the absorption rate of a metabolite *m* from the pool, D_*m*_ is the amount of metabolite *m* in the pool, *A*_*m*_ is the bioavailability of *m*, and ${t}_{{\mathrm{max}}}^{m}$ is the time-of-maximum appearance rate of metabolite *m* in the blood. For each time step, before optimising the model, the amount determined by *U*_*m*_(*t*) was added to the current blood metabolite level. All the blood metabolite amounts were then converted back to concentrations by multiplying them by a blood volume of 5 L. Note: in the fasting condition, the gut is empty and *U*_*m*_(*t*) is equal to 0.

To reflect the required adaptation of the system to the new blood metabolite levels, a Michaelis–Menten equation (Eq. (22)), dependent on the deviation of the current blood metabolite concentration from the baseline [*S*_*m*_], was used to limit the maximum uptake rate of the food metabolites from the blood stores to the tissues:

22 $${v}_{m}=\frac{{v}_{{\mathrm{max}}}^{m}\cdot [{S}_{m}]}{{k}_{M}^{m}+[{S}_{m}]}$$

23 $$[{S}_{m}]={[{S}_{m}]}_{{\mathrm{current}}}-{[{S}_{m}]}_{{\mathrm{basal}}}$$

where ${[{S}_{m}]}_{{\mathrm{current}}}$ is the respective blood metabolite concentration after the absorption of meal metabolites, and [S_*m*_]_basal_ is the basal blood metabolite concentration. Due to numerical errors, [*S*_*m*_] might become negative (in the order of 10^−7^. If this occurred, then *v*_*m*_ was not calculated and was set to 0.

For each metabolite *m* considered in the food pool, the parameters ${t}_{{\mathrm{max}}}^{m}$, ${v}_{{\mathrm{max}}}^{m}$, and ${k}_{M}^{m}$ were fitted simultaneously to the average blood metabolite profile from Milan et al.^21^. The optimization was performed using the lsqnonlin function from the Optimization Toolbox in MATLAB.

The upper bounds of the fitting parameters were set in the following way: the ${t}_{{\mathrm{max}}}^{m}$ upper bounds were set to 200 min, the ${v}_{{\mathrm{max}}}^{m}$ upper bounds were set to 1000 μmol per min, and the ${k}_{M}^{m}$ upper bounds were set to 1000 μmol per L. As cystein, and tryptophan were not measured, no parameter fit for these two amino acids was performed. Thus, for cystein, and for tryptophan, the serine and the phenylalanine parameter values were used, respectively. A table containing all the metabolite amounts for each meal and the respective fitted parameters values is available in Supplementary Table 10. The results of the model fit can be found in Supplementary Fig. 15.

### Simulating exercise

To simulate exercise at different intensity levels, it is necessary to represent the exercise in the model. Since exercise mainly consists of energy expenditure, exercise was simulated by varying the ATP demand in the muscle. First, we investigated by how much the ATP demand in the muscle would need to be increased to reach 90% of the maximal O_2_ consumption in the model. This was achieved by simulating exercise by increasing the ATP demand in the muscle until the expected O_2_ consumption was observed. Thus, by multiplying the muscle basal ATP demand by a factor of 42, an exercise eliciting the consumption of 90% O$_{2,\ \max }$ was observed. The other ATP demands were determined, by assuming that the linear increase of ATP demand in the muscle, leads to the linear increase in O_2_ consumption. I.e. to determine by how much the ATP consumption in the muscle needed to be increased to reach 80% O$_{2,\ \max }$, the following calculation was done: (42 ⋅ 80)/90 = 37.3. Different experimental setups were extracted from literature, and simulated in the multi-tissue model. The exercise was performed, for all exercise simulations, after 12 h of fasting to represent as close as possible the experimental setups extracted from literature, with the exception of when a meal was given prior to the exercise in the setup of interest. In this case, the meal was given after 12 h of fasting, and the exercise was simulated 1 h after the meal was ingested.

Simulation including steady exercise at different intensity levels during fasting:

- Simulation 1: 90 min of 40% O$_{2,\ \max }$ exercise followed by 3 h resting^29^.
- Simulation 2: 60 min of 60% O$_{2,\ \max }$ exercise followed by 3 h resting^29^.

Simulation including steady and incremental exercise at different intensity levels during fasting:

- Simulation 3: 5 min of 50% O$_{2,\ \max }$ exercise followed by 1 h 55 of 70% O$_{2,\ \max }$ exercise. After 30 min resting, the simulation of incremental exercise started. 10 min exercise at 70% O$_{2,\ \max }$, 10 min at 80% O$_{2,\ \max }$, and 10 min at 90% O$_{2,\ \max }$^30^ were simulated.

The following simulations were performed for a low fat meal:

- Simulation 4: 60 min of 55% O$_{2,\ \max }$ exercise followed by 30 min rest. After resting the meal was applied, and the model simulated for another 150 min^23^.
- Simulation 5: the meal was applied. After 60 min resting, 60 min of 55% O$_{2,\ \max }$ exercise was simulated, followed by 180 min resting^23^.

An additional simulation was performed to compare the effect of exercising, or resting in the fed state:

- Simulation 6: the meal was applied, and no exercise was simulated.

The fatty acid oxidation was calculated in multiple steps. First, the individual fatty acid oxidation reactions were collected. Depending on the reaction, more than one molecule of acetyl-coa could be produced. The acetyl-coa coefficient was extracted, and each flux was multiplied by the respective coefficient. The fluxes were summed up, and the AUC calculated.

Multiple energy precursors can be used to provide energy during exercise to the muscle: (1) free fatty acids absorbed from the blood; (2) other fat sources, representing the sum of blood TAG absorption and the degradation of TAG stores in muscle; (3) blood glucose absorbed; and (4) glycogen degraded in muscle.

The calculation of the availability of each of these sources for ATP production was performed in several steps:

- The AUC of the flux of the different energy precursors for the muscle was calculated.
- TAG and glycogen were broken down into free fatty acids, and glucose molecules, respectively. TAG contains 3 fatty acids, thus the AUC value of "other fat sources" was multiplied by 3. Glycogen degradation produces 8 molecules of glucose and 3 molecules of glucose-1-phosphate, thus the AUC value of muscle glycogen degradation was multiplied by 11.
- The degradation of one molecule of glucose, and an average free fatty acid leads to the production of around 31, and 106 ATP molecules^57^, respectively. Therefore, to obtain the ATP contribution of each energy source, the AUC values of glucose, and glycogen-derived glucose were multiplied by 31, and those from free fatty acids, and TAG-derived free fatty acids were multiplied by 106.

### Mapping of IEMs onto the multi-tissue metabolic model

A list of IEMs was collected from several published studies^3,32,58,59^ to simulate IEMs in the multi-tissue model. These studies were selected, as literature biomarkers were included in the papers. The IEMs were mapped to a list of IEMs impaired genes/enzymes^60^, and the respective genes extracted. IEMs were simulated only if the impaired genes directly affected a metabolic reaction of the multi-tissue model (e.g., an IEM was only simulated if the function deleteModelGenes from the COBRA aToolbox indicated an effect of the gene knockout).

In total, 65 IEMs were simulated under different conditions (in the fasting state, after a low fat meal, and after a high fat meal). The simulation of IEMs was performed by setting the targeted reaction(s) fluxes to 0 (i.e., upper and lower bounds were fixed to 0, representing a 100% inactivation). In addition, 100 "individual" models for each condition were simulated (fasting state, low fat meal, and high fat meal). For each of these models, the flux bounds of all the flexible constraints were randomly decreased, or increased to mimic the metabolism differences between individuals. The random change ranged from 0 to 10%.

In total, 498 models were simulated:

- 65 IEMs ⋅ 3 conditions
- 100 “Individual” healthy models ⋅ 3 conditions
- Average healthy model ⋅ 3 conditions

### Predicting outlier data points as potential metabolic biomarkers

Metabolic biomarkers are metabolites whose levels are outside the healthy range, either above or below. Thus, one method which might help in their identification is by predicting which metabolite levels might be outliers. This was achieved by performing several steps: (1) preprocessing, (2) “outliers” identification, and (3) filtering.

Data was pre-processed as following:

- For each metabolite, the minimum amount among all simulations was calculated. For some metabolites, the blood amount became negative during the simulation, as well as the urine fluxes. These negative values were in the order of 10^−7^, and occurred because of numerical errors during the optimization process.
- For those blood metabolites where a negative amount was found during the simulation, the absolute value of the negative change was added to all the simulations in order to obtain only positive blood amounts.
- For the urine metabolites, any negative flux was replaced by zero.

### Outliers identification

After the preprocessing, the AUC of the blood level/urine excretion flux over time was calculated for each metabolite in each condition, and hierarchical clustering analysis was performed to identify potential biomarkers. This was achieved by using the MATLAB cluster function from the Statistics and Machine Learning Toolbox. Default parameters were selected, with the exception of the linkage measure which was set to single. The silhouette function, from the same toolbox, was used to identify the best number of clusters (ranging from 1 to 3), for each metabolite among all conditions (*n* = 498). The number of clusters represents the following: a cluster number of 1 indicates that the metabolite was inside the healthy range for all the conditions; a cluster number of 2 indicates that at least one metabolite level was outside the healthy range (one metabolite level was decreased or increased when compared to the healthy range); finally, a cluster number of 3 indicates i.e., that in some conditions the metabolite level was decreased, while in others it was increased.

### Final list of biomarkers

After extracting the potential list of biomarkers, two steps of filtering were performed.

- In the first step, metabolites with AUC larger or smaller than the healthy range were kept, and the others removed from the list. The healthy range is defined as the minimum and maximum AUC values obtained from the simulations of the healthy models (*n* = 303).
- In the last step, metabolites which were only identified as biomarkers in the fasting condition were removed. In general, the clinical symptoms of amino acidopathies, e.g., phenylalanine, will manifest when the affected individuals ingest foods rich in the non metabolizable amino acid, e.g., phenylalanine, leading to its accumulation in the blood.

A score was assigned to each metabolite. This score represents the total number of conditions (fasting, low fat meal and high fat meal) that a biomarker was identified in. The score value ranged from 0 to 3. A score of 0 represents non biomarkers (metabolites in the healthy range); a score of 1 represents biomarkers identified in only one condition (i.e., after a low fat meal, or after a high fat meal); a score of 2 represents biomarkers identified in two conditions; and a score of 3 represents biomarkers which were identified in all 3 conditions. Furthermore, a sign was added to the score value to represent an increase (plus sign), or a decrease in the biomarker (minus sign) level, when compared to the healthy range.

### Software

The micro array data processing was performed with R version 3.2.0. All the simulations were performed with MATLAB 2014b 32bit using ILOG Cplex 32 bit (cplex1261). The model manipulation, and simulation was performed using the COBRA Toolbox (v2)^61^. The figures were either created with R version 3.4.0 or MATLAB 2014b 32bit.

## Supplementary information

Supplementary Information

Supplementary Data 1

Supplementary Data 2

Supplementary Data 3

Supplementary Data 4

Supplementary Data 5

Supplementary Data 6

Supplementary Data 7

## Data Availability

The transcriptomic’s data used to reconstruct the tissue models is publicly available on the GEO database (accession numbers in Supplementary Table 2). The meal composition, and the blood metabolite profiles were kindly provided upon request by Prof. David Cameron-Smith^21^.

## Code availability

The code used to reconstruct and simulate the model is available upon request to the corresponding author.
