# Supplementary material for: A dynamic multi-tissue model to study human metabolism
Source: NPJ Syst Biol Appl. 2021 Jan 22;7:5. doi: 10.1038/s41540-020-00159-1 (PMC7822846; doi:10.1038/s41540-020-00159-1)
Supplement: Supplementary file 1 — Supplementary Information [file 41540_2020_159_MOESM1_ESM.pdf]

A dynamic multi-tissue model to study human metabolism

Patricia Martins Conde[1,2], **Thomas Pfau**[1], **Maria Pires Pacheco**[1], **and Thomas Sauter**[1,*]

[1]Department of Life Sciences and Medicine, University of Luxembourg, Luxembourg
[2]Megeno S.A., Luxembourg
[*]Corresponding author: thomas.sauter@uni.lu

ABSTRACT

Metabolic modelling enables the study of human metabolism in healthy and in diseased conditions, e.g. the prediction of new drug targets and biomarkers for metabolic diseases. To accurately describe blood and urine metabolite dynamics, the integration of multiple metabolically active tissues is necessary. We developed a dynamic multi-tissue model, which recapitulates key properties of human metabolism at the molecular and physiological level based on the integration of transcriptomics data. It enables the simulation of the dynamics of intra- and extra-cellular metabolites at the genome scale. The predictive capacity of the model is shown through the accurate simulation of different healthy conditions (i.e. during fasting, while consuming meals or during exercise), and the prediction of biomarkers for a set of Inborn Errors of Metabolism with a precision of 83%. This novel approach is useful to prioritize new biomarkers for many metabolic diseases, as well as for the integration of various types of personal omics data, towards the personalized analysis of blood and urine metabolites.

List of Figures

Supplementary Figure 1 . . . 3
Supplementary Figure 2 . . . 4
Supplementary Figure 3 . . . 5
Supplementary Figure 4 . . . 6
Supplementary Figure 5 . . . 7
Supplementary Figure 6 . . . 8
Supplementary Figure 7 . . . 9
Supplementary Figure 8 . . . 10
Supplementary Figure 9 . . . 11
Supplementary Figure 10 . . . 12
Supplementary Figure 11 . . . 13
Supplementary Figure 12 . . . 14
Supplementary Figure 13 . . . 15
Supplementary Figure 14 . . . 16
Supplementary Figure 15 . . . 17

List of Tables

Supplementary Table 1 . . . 18
Supplementary Table 2 . . . 19
Supplementary Table 3 . . . 20
Supplementary Table 4 . . . 20
Supplementary Table 5 . . . 20
Supplementary Table 6 . . . 21
Supplementary Table 7 . . . 22
Supplementary Table 8 . . . 22
Supplementary Table 9 . . . 22
Supplementary Table 10 . . . 23

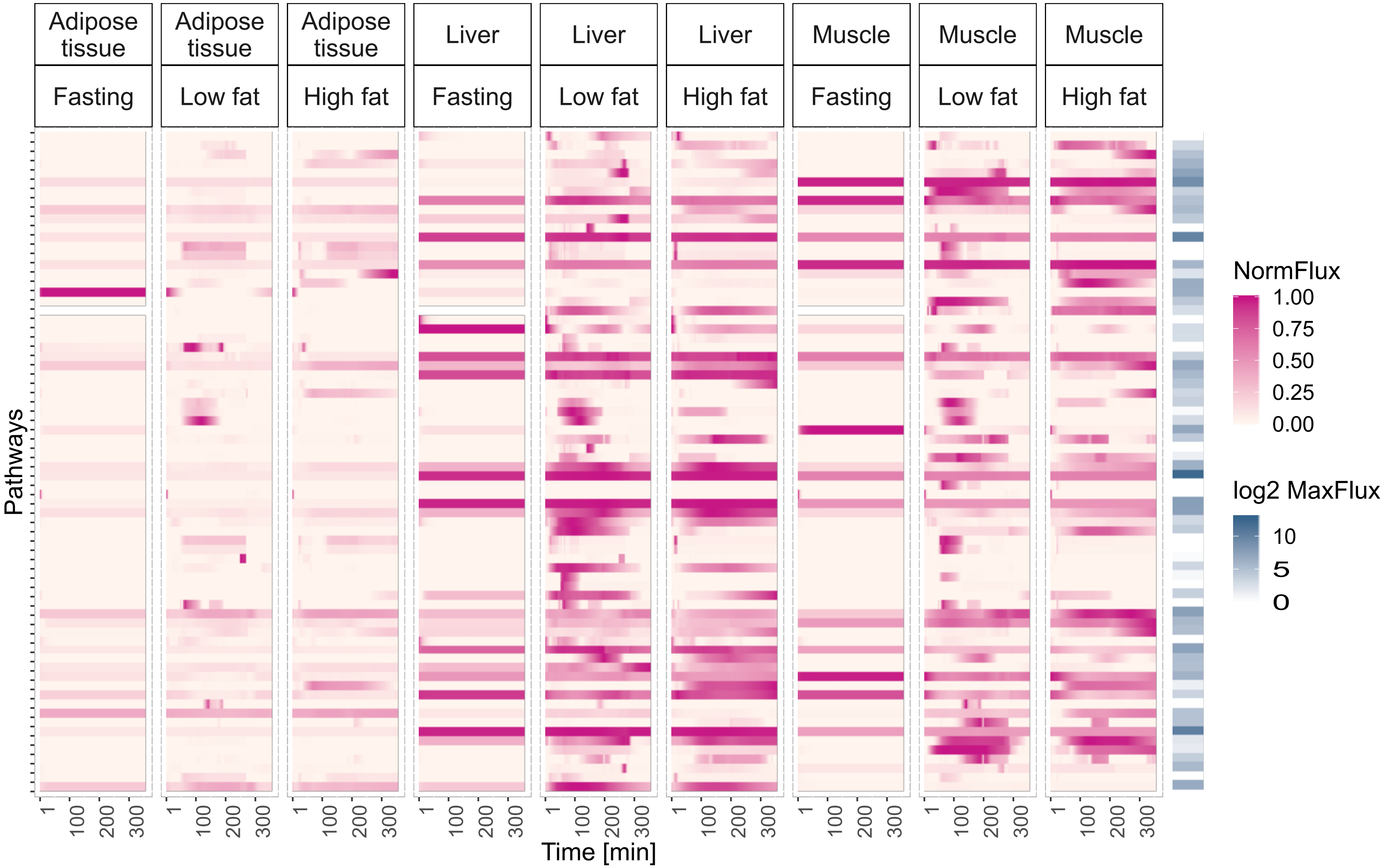

Supplementary Figure 1. Active metabolic pathways in different conditions. Each flux in a subsystem was normalized to the maximum flux of that subsystem.

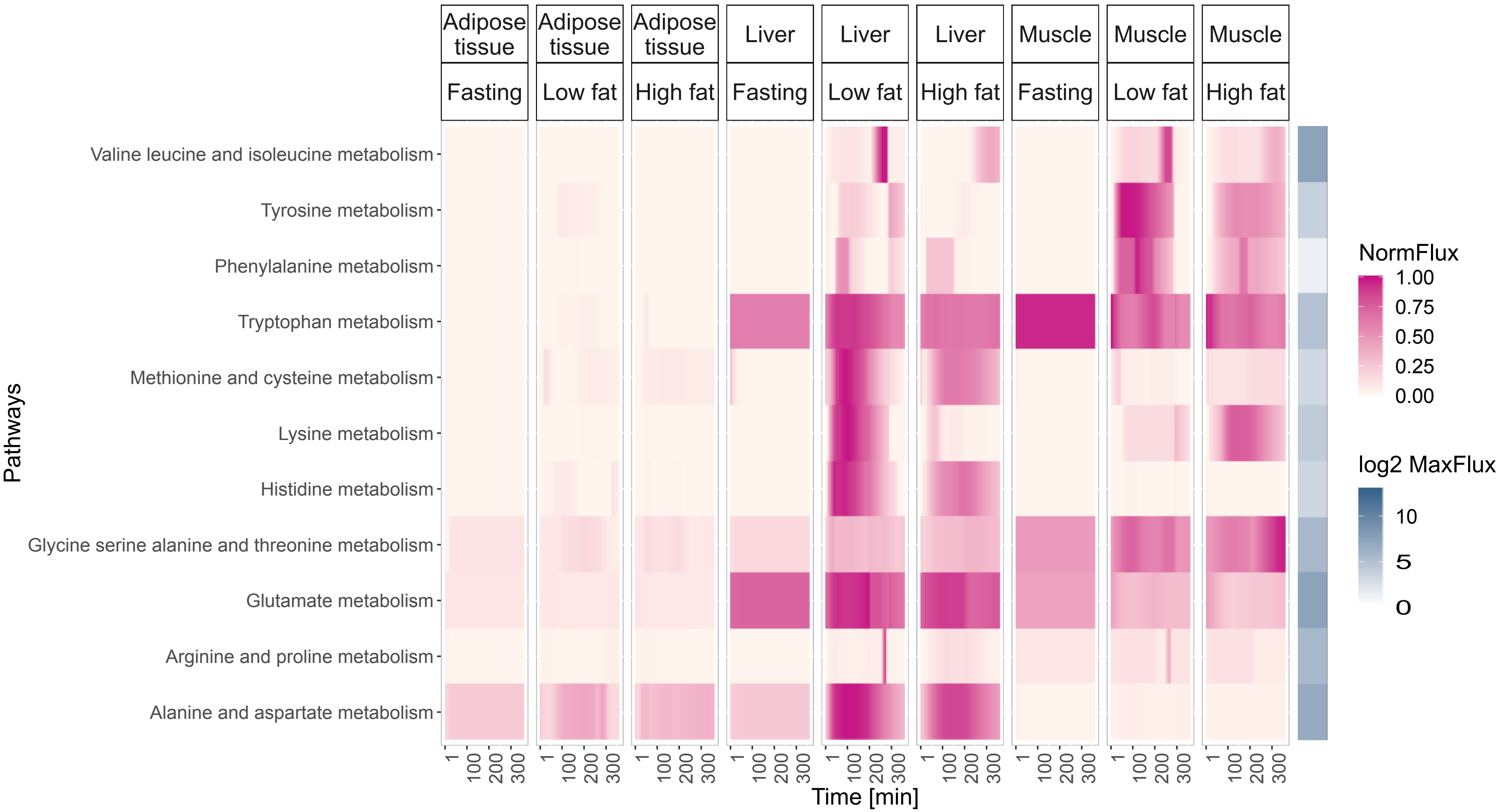

Supplementary Figure 2. Amino acid pathways usage during different conditions. Each flux in a subsystem was normalized to the maximum flux of that subsystem.

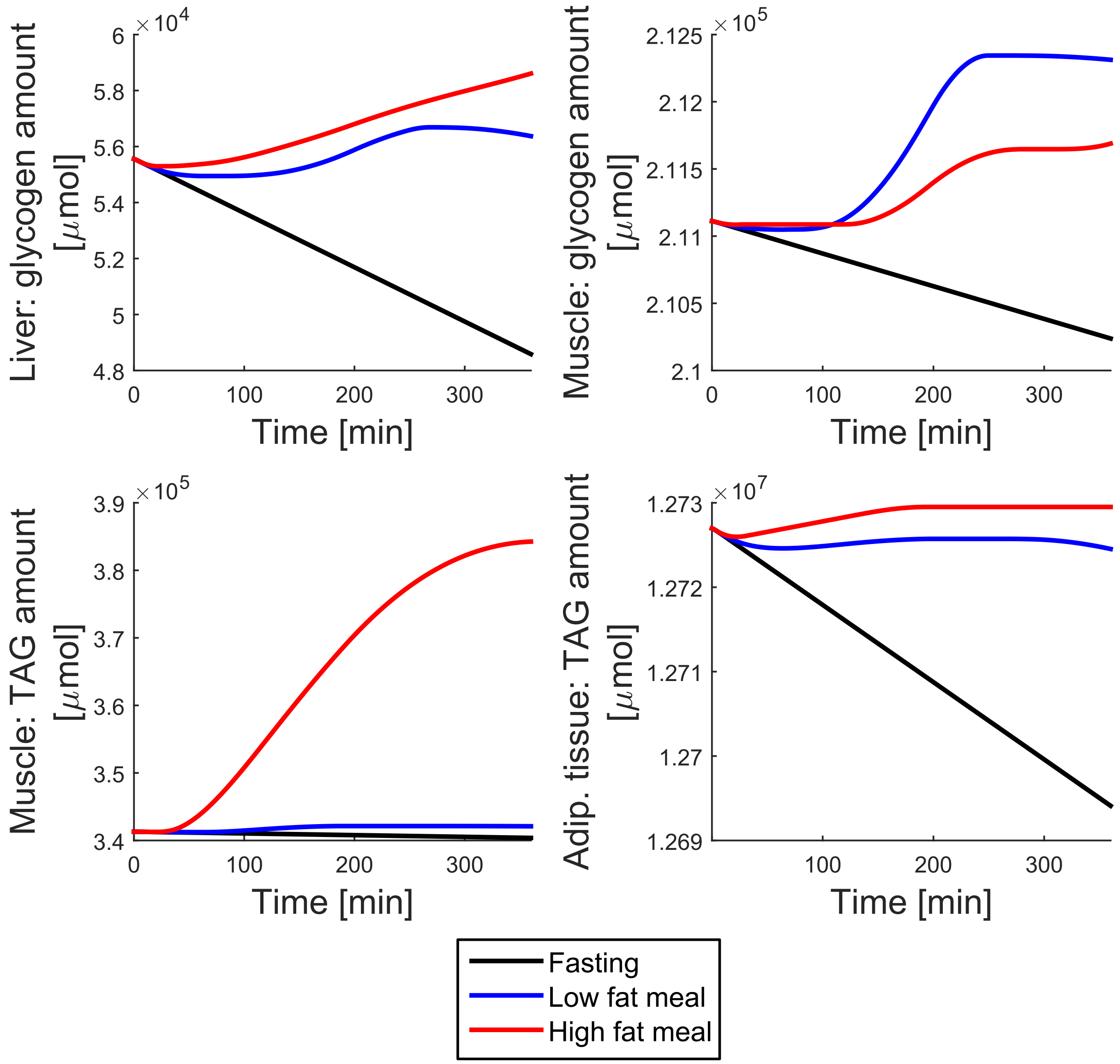

Supplementary Figure 3. Comparison of the effect of different conditions on the internal energy stores in different tissues. The meals led to an increased storage of TAG in the adipose tissue, and in the muscle, and glycogen storage in the liver and the muscle. The storage of TAG became more pronounced, with the increased amount of fat in the meal. The glycogen storage was larger in the liver following a high fat meal, while the low fat meal elicited an increased glycogen storage in the muscle.

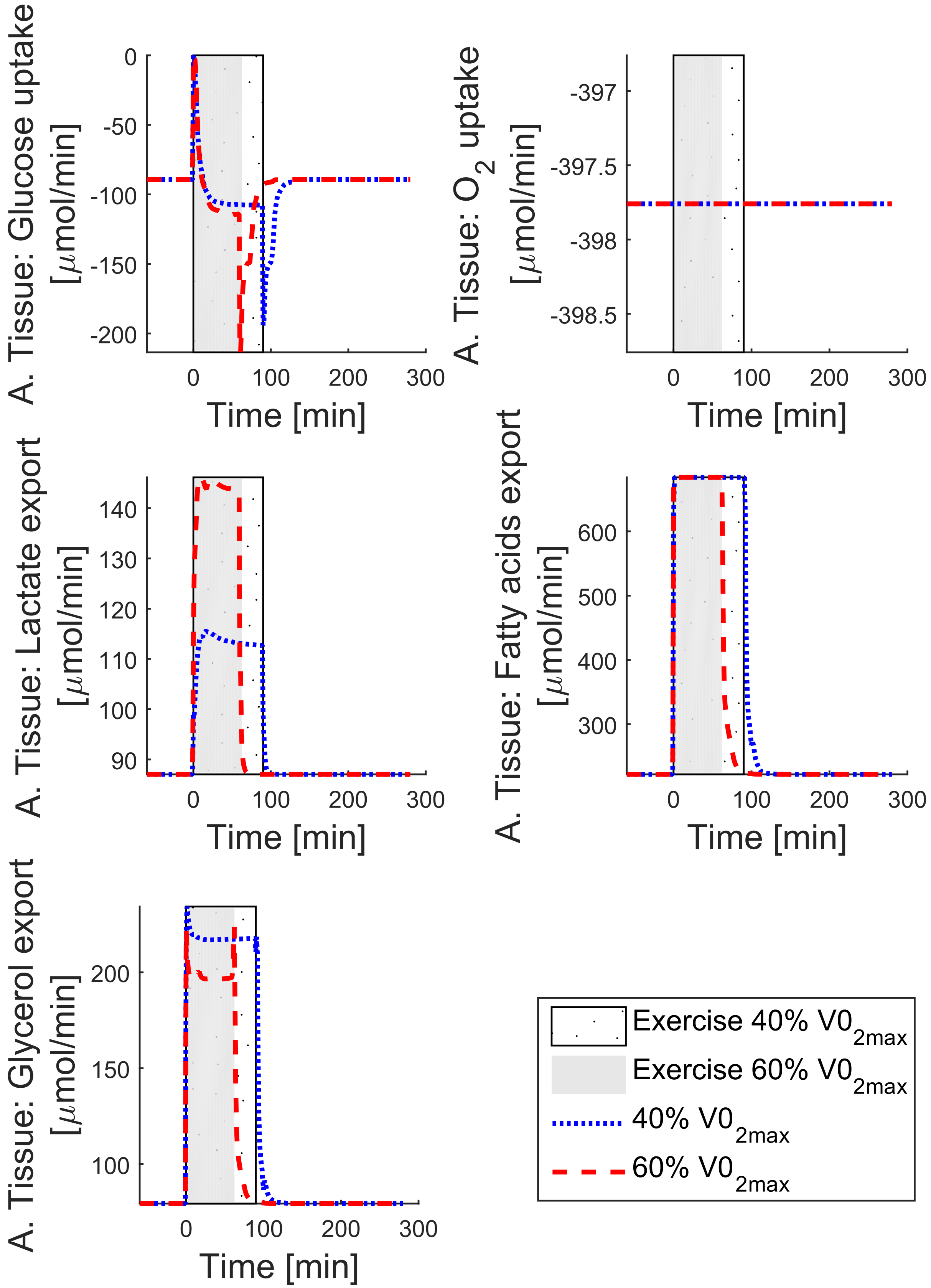

Supplementary Figure 4. Effect of exercising on adipose tissue fluxes. In blue is depicted the model prediction of exercising at 40% $O_{2,\ max}$, and in red is depicted the model prediction of exercising at 60% $O_{2,\ max}$. The fatty acids flux correspond to the sum of the all fatty acids fluxes in the adipose tissue model.

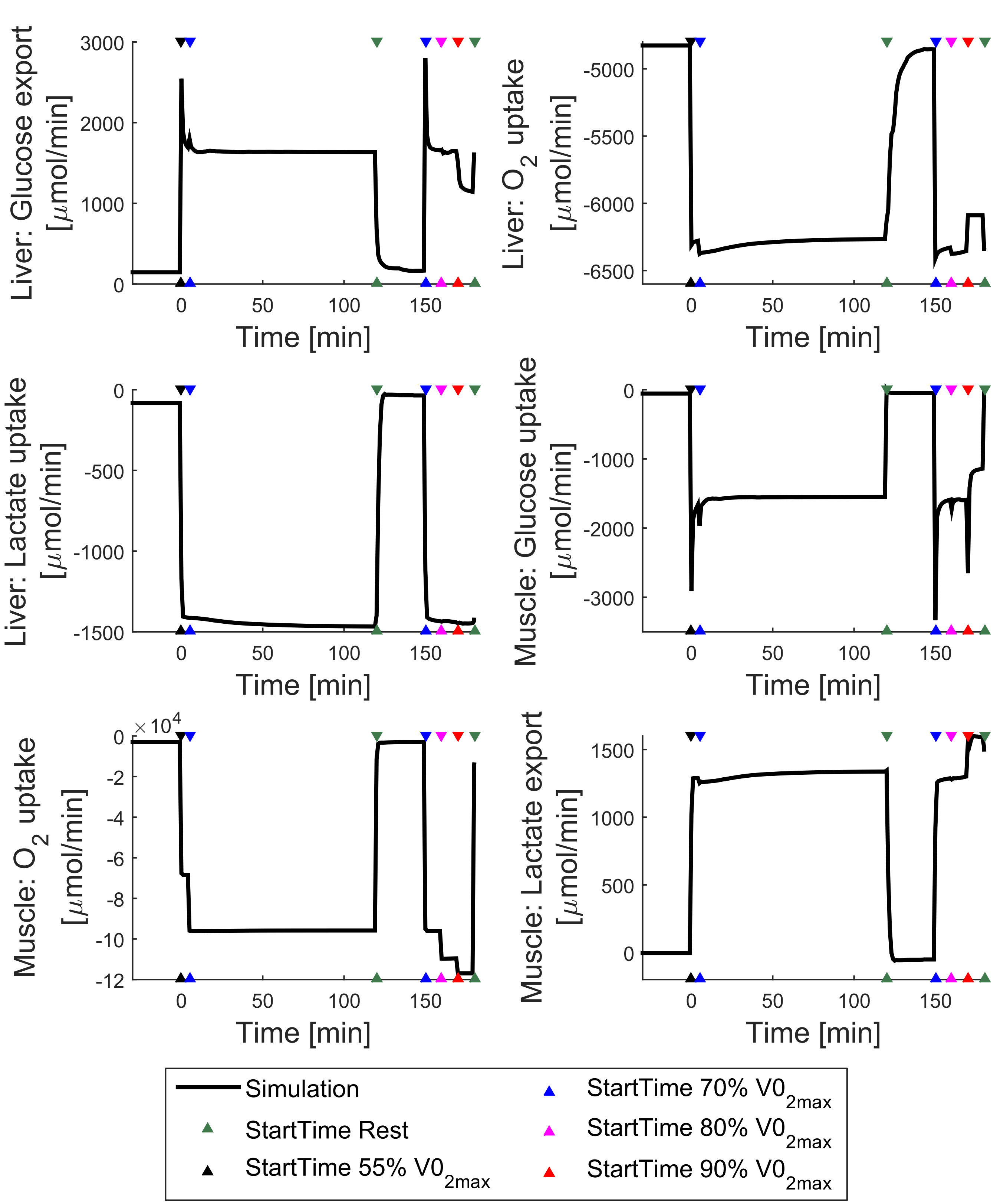

Supplementary Figure 5. Effect of steady and incremental exercising on muscle, and on liver fluxes. Simulation 3: 5 min of 50% $O_{2,\,max}$ exercise followed by 1h55 of 70% $O_{2,\,max}$ exercise (steady exercise). After 30 min resting, incremental exercise was simulated. Each phase of the incremental exercise was simulated for 10 min (10 min exercise at 70% $O_{2,\,max}$, 10 min at 80% $O_{2,\,max}$, and 10 min at 90% $O_{2,\,max}$).

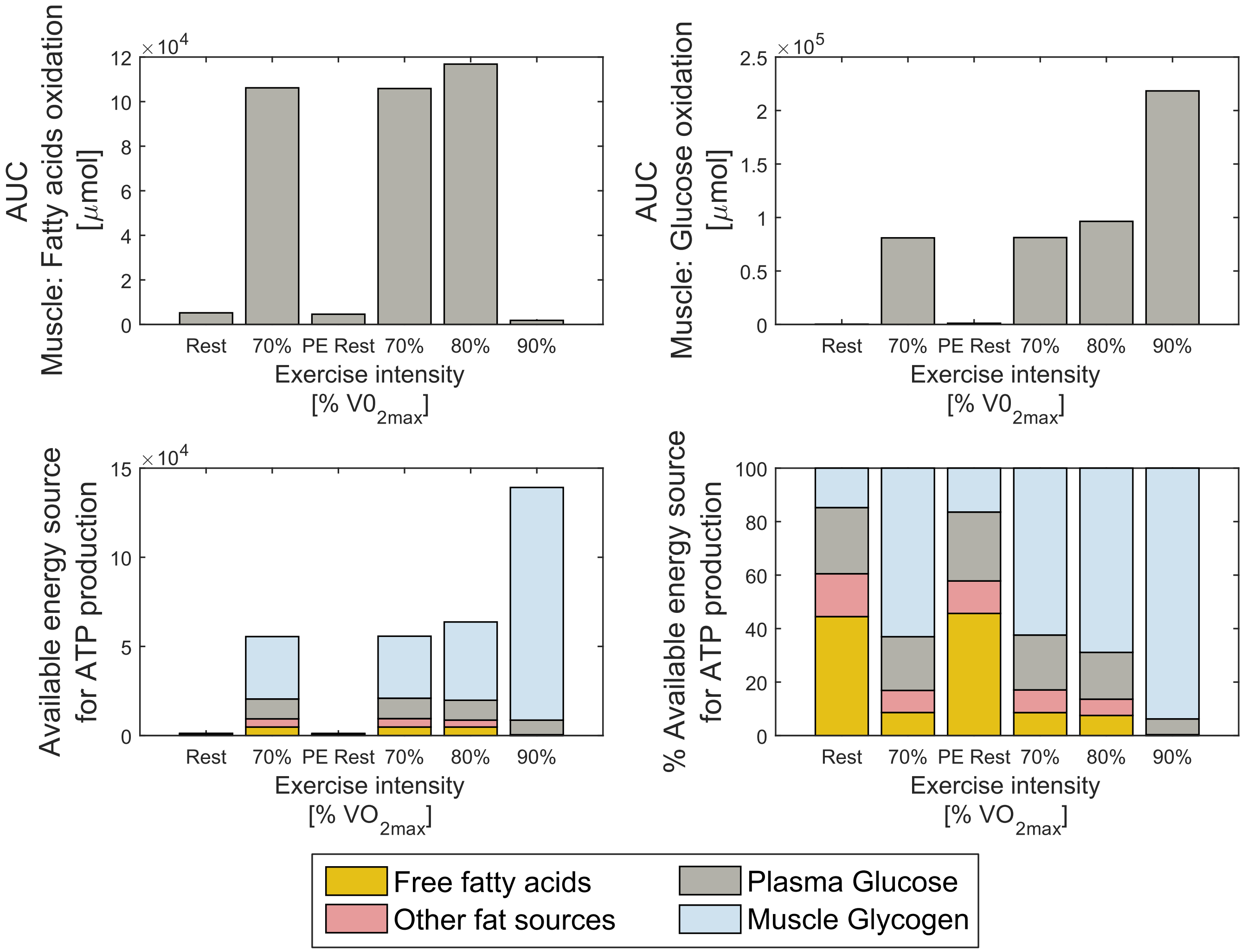

Supplementary Figure 6. Incremental exercise affects the usage of each energy source in the muscle. The incremental exercise was simulated during 10 minutes each. To calculate the AUC, only 8 minutes of each condition were taken into consideration. During the first two minutes, the model needed to adapt to the changing condition. The steady exercise was performed for 1 hour and 55 minutes, but to make the AUC values comparable, only the 8 minutes, after 2 minutes of adaption, were taken into consideration. For the calculation of the resting AUC, only the last 8 minutes preceding the start of the exercise were used. The glucose oxidation represents the flux through the PDHm in the muscle, where acetyl-coa is produced from pyruvate. The fatty acid oxidation and the availability of each source for ATP production were calculated, as described in the methods. Other fat sources represent TAG uptake in the muscle, and the usage of the muscle internal TAG stores. Abbreviations: PE Rest=Post exercise resting state.

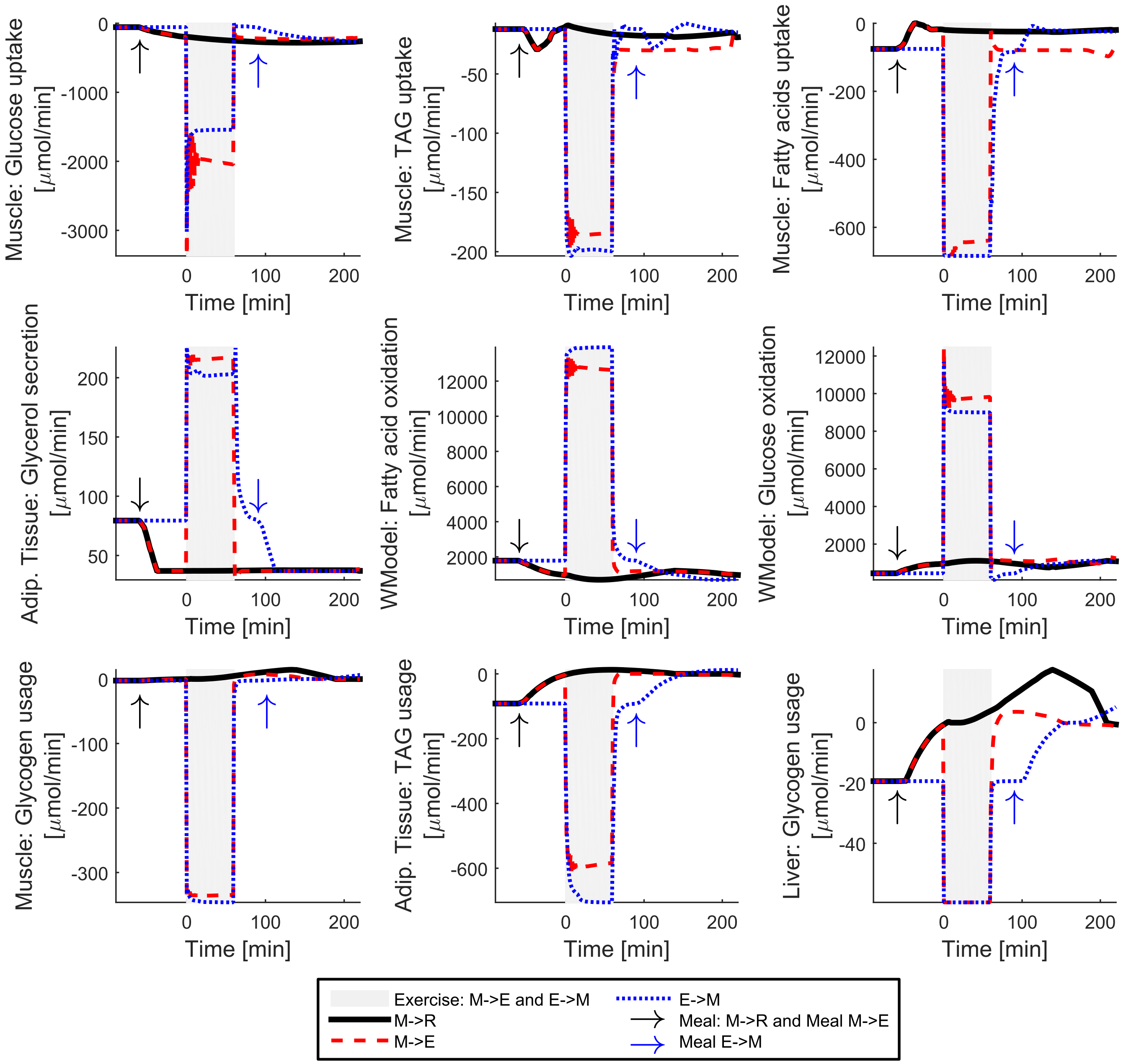

Supplementary Figure 7. Effect of a low fat meal and exercise on specific fluxes, and tissue stores usage. Three different conditions were simulated to compare their effect on the model. The predicted effect of a low fat meal on specific components of the model (M $\rightarrow$ R) is shown in black. The predicted effect of consuming a low meal before performing 60 min of exercise (M $\rightarrow$ E) is shown in red. The predicted effect of performing 60 min exercise before consuming a low fat meal (E $\rightarrow$ M) is represented in blue. The arrow represents the time point where the meal ingestion started, while the grey background represents the time interval during which the exercise was simulated. The glucose oxidation represents the flux through the PDHm in the three tissues, where acetyl-coa is produced from pyruvate. The fatty acid oxidation was determined as described in the Methods section. Abbreviations: WModel=Whole-model; Adip. Tissue=Adipose tissue.

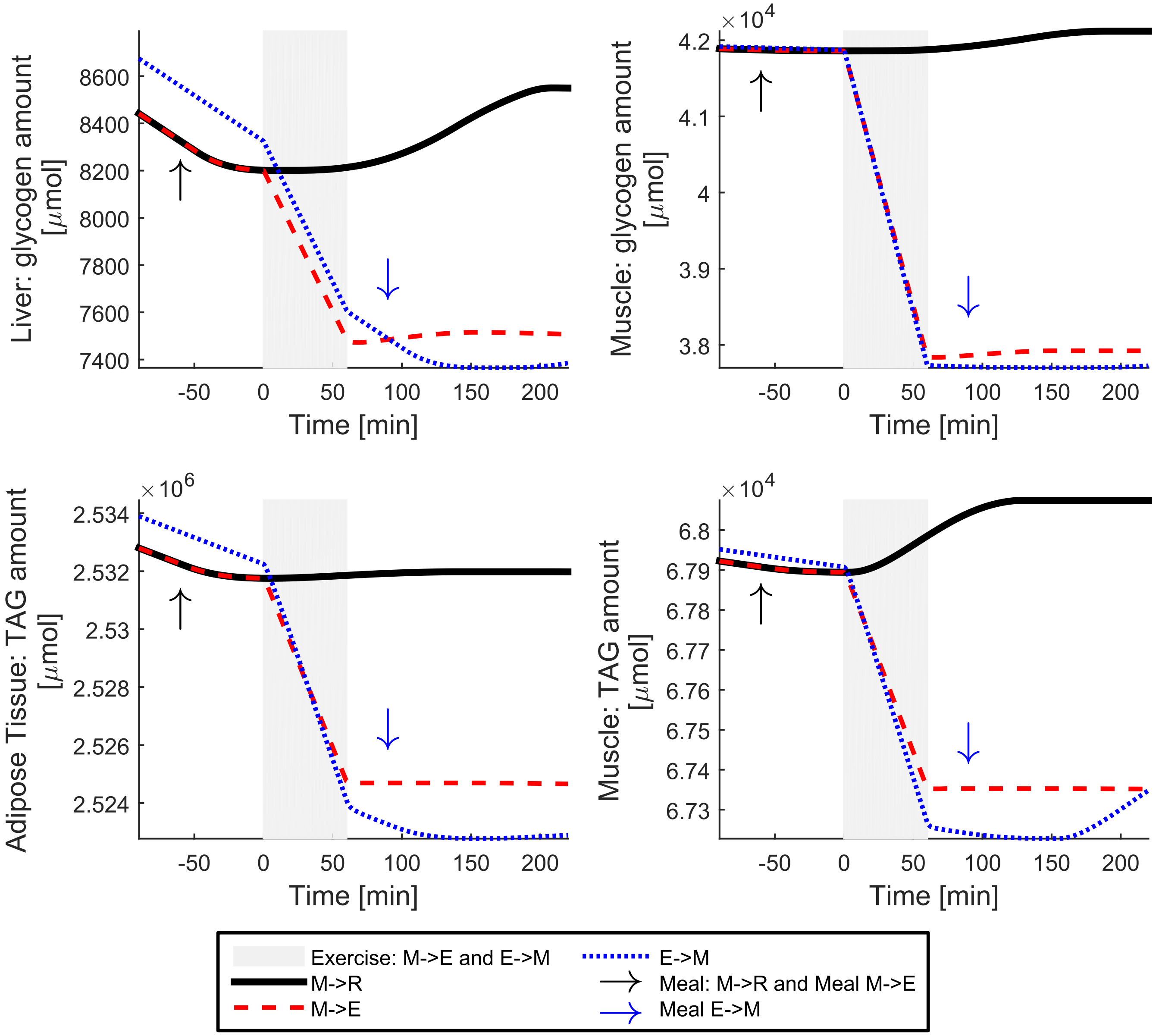

Supplementary Figure 8. Effect of a low fat meal and exercise on the internal energy stores. Three different conditions were simulated to compare their effect on the internal energy storage level. The predicted effect of a low fat meal on specific components of the model (M $\rightarrow$ R) is shown in black. The predicted effect of consuming a low fat meal before performing 60 min of exercise (M $\rightarrow$ E) is depicted in red. The predicted effect of performing 60 min exercise before consuming a low fat meal (E $\rightarrow$ M) is shown in blue. The arrow represents the time point where the meal ingestion started, while the grey background represents the time interval during which the exercise was simulated. Abbreviations: StartT=Starting time, StopT=Stopping time, WModel=Whole-model; Adip. Tissue=Adipose tissue.

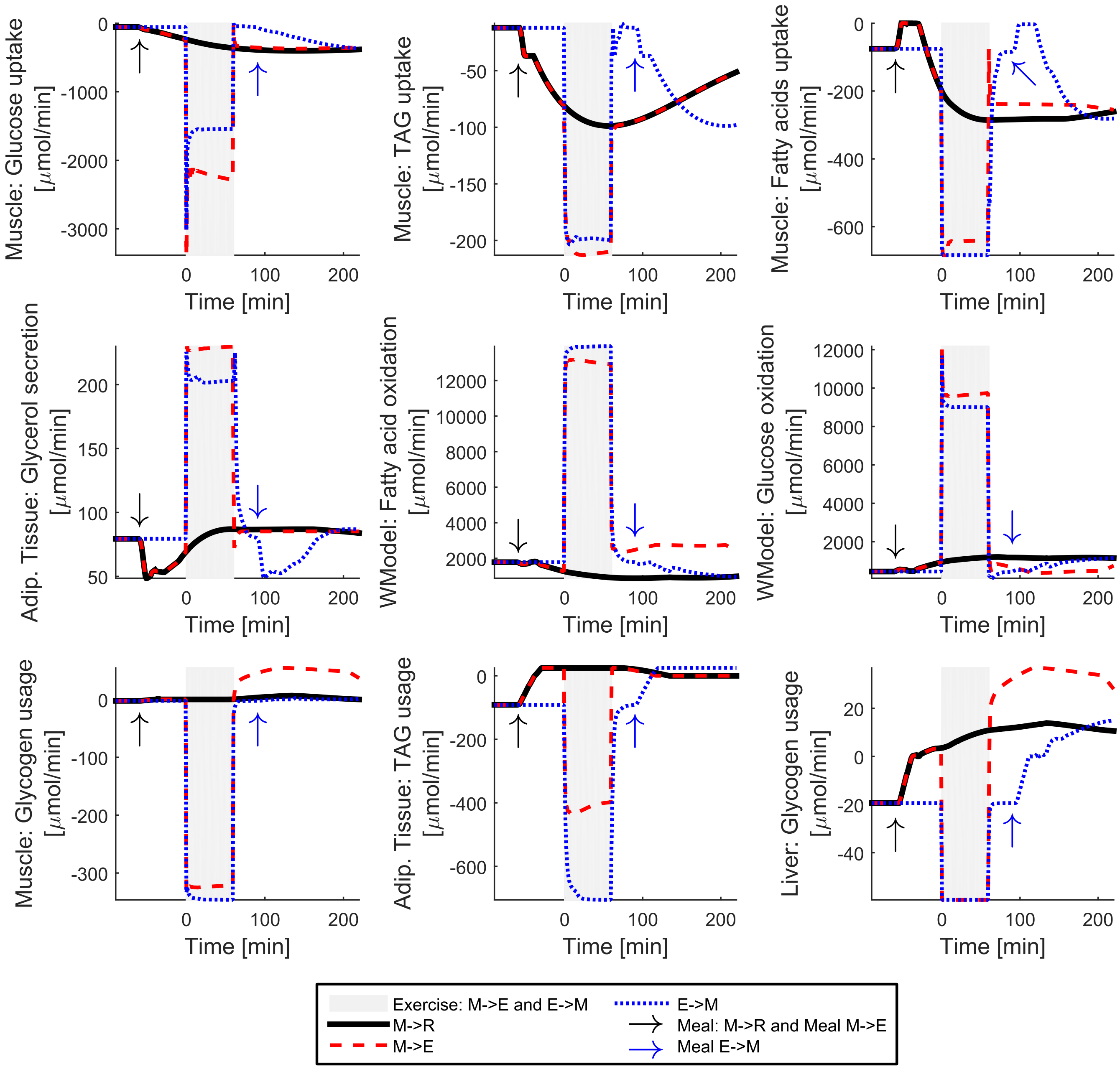

Supplementary Figure 9. Effect of a high fat meal and exercise on specific fluxes and tissue stores usage. Three different conditions were simulated to compare their effect on the model. The predicted effect of a high fat meal on specific components of the model (M $\rightarrow$ R) are shown in black. The predicted effect of eating a high meal before performing 60 min of exercise (M $\rightarrow$ E) are depicted in red. The predicted effect of performing 60 min exercise before consuming a high fat meal (E $\rightarrow$ M) is shown in blue. The arrow represents the time point where the meal ingestion started, while the grey background represents the time interval during which the exercise was simulated. The glucose oxidation represents the flux through the PDHm in the three tissues, where acetyl-coa is produced from pyruvate. The fatty acid oxidation was determined as described in the Methods section. Abbreviations: WModel=Whole-model; Adip. Tissue=Adipose tissue.

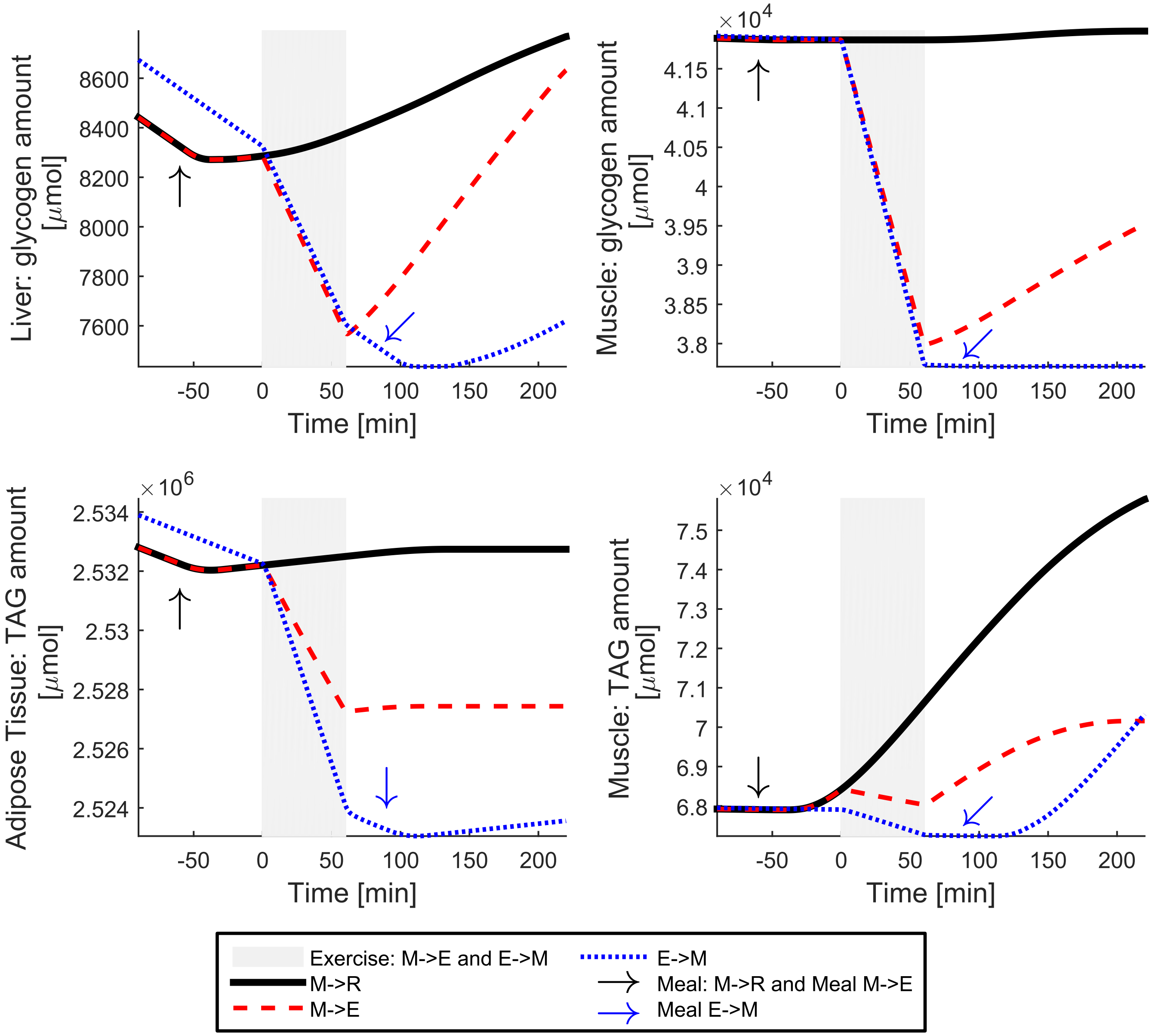

Supplementary Figure 10. Effect of a high fat meal and exercise on the internal energy stores. Three different conditions were simulated to compare their effect on the internal energy storage level. The predicted effect of a high fat meal on specific components of the model (M $\rightarrow$ R) is represented in black. The predicted effect of consuming a high fat meal before performing 60 min of exercise (M $\rightarrow$ E) is shown in red. The predicted effect of performing 60 min exercise before consuming a high fat meal (E $\rightarrow$ M) is depicted in blue. The arrow represents the time point where the meal ingestion started, while the grey background represents the time interval during which the exercise was simulated. Abbreviations: StartT=Starting time; StopT=Stopping time; WModel=Whole-model; Adip. Tissue=Adipose tissue.

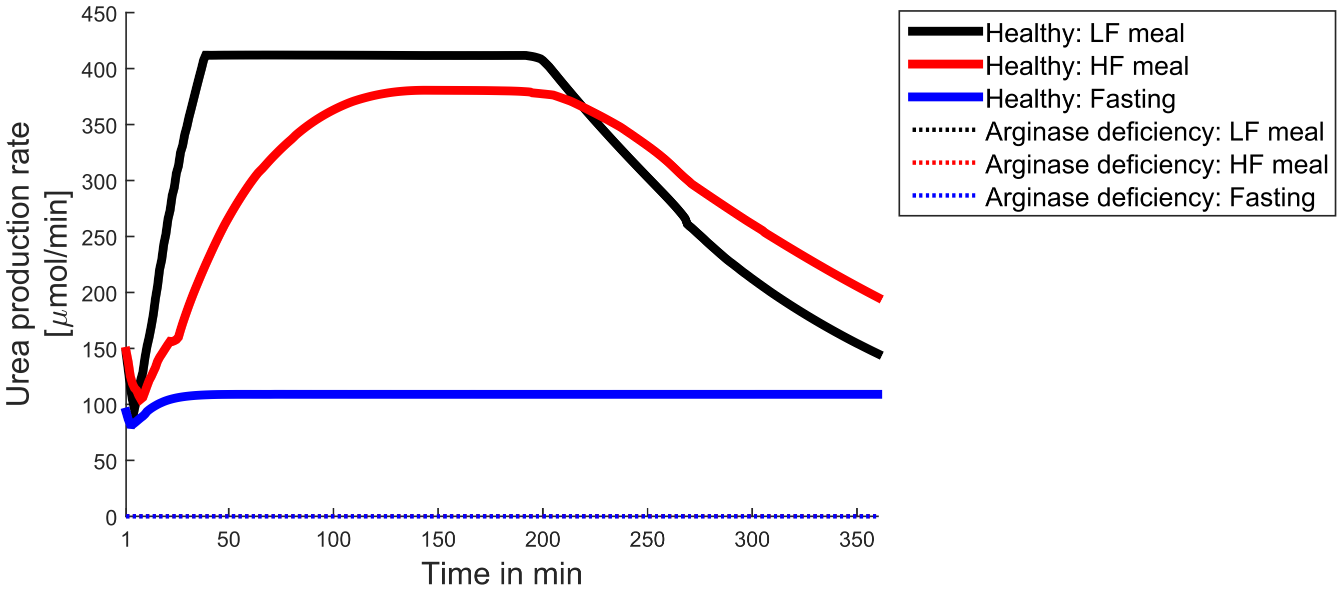

Supplementary Figure 11. Effect of *Arginase1* knockout on the urea production rate. Fasting, and different fed conditions were simulated to predict the effect of *Arginase1* knock-out on urea production. The urea production curbs of the three arginase deficiency simulations are overlapping. Therefore, only the fasting simulation is visible. Abbreviations: LF meal=Low fat meal; HF meal=High fat meal.

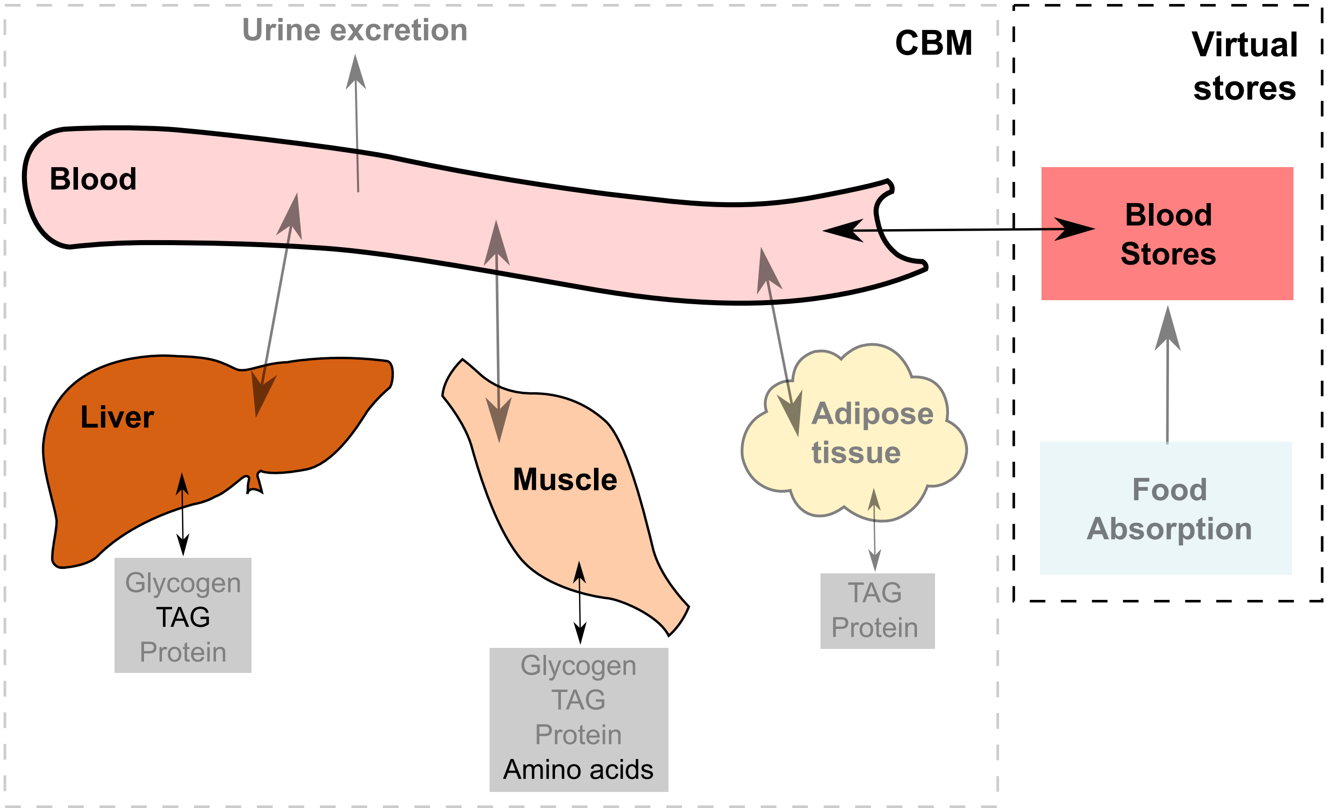

Supplementary Figure 12. Maintenance of homeostasis of specific metabolites. Metabolite amounts present in the blood stores, and the amino acids in the muscle store are used to constrain the model. The liver TAG store is initialized by setting its amount to 0 μmol. This store was used in the same way as the stored blood metabolites, and stored amino acids in the muscle. The main goal of these constraints were to maintain the blood metabolites, the muscle amino acids and the liver TAG stores at the basal levels. This constraint was used to avoid to store, or deplete metabolites, if it wasn't necessary. After the ingestion of a meal, instead of allowing the metabolites to accumulate in the blood, the model is forced to use them, to return the blood levels to the basal condition as soon as possible. At the same time, in case of an enzyme impairment, this constraint still allows, and give some flexibility to accumulate potential by-products in the blood or in the muscle. It is important to mention here, that stores levels are not allowed to become negative, since hard constraints were set to avoid it. Additionally, while blood levels could get above, or below the healthy level, the muscle amino acids couldn't. This ensured that in case of enzyme impairment, blood levels might get larger, or lower than the healthy level, while preventing the usage or the storage of amino acids in the muscle above or below the healthy levels. Abbreviations: TAG=Triacylglycerol.

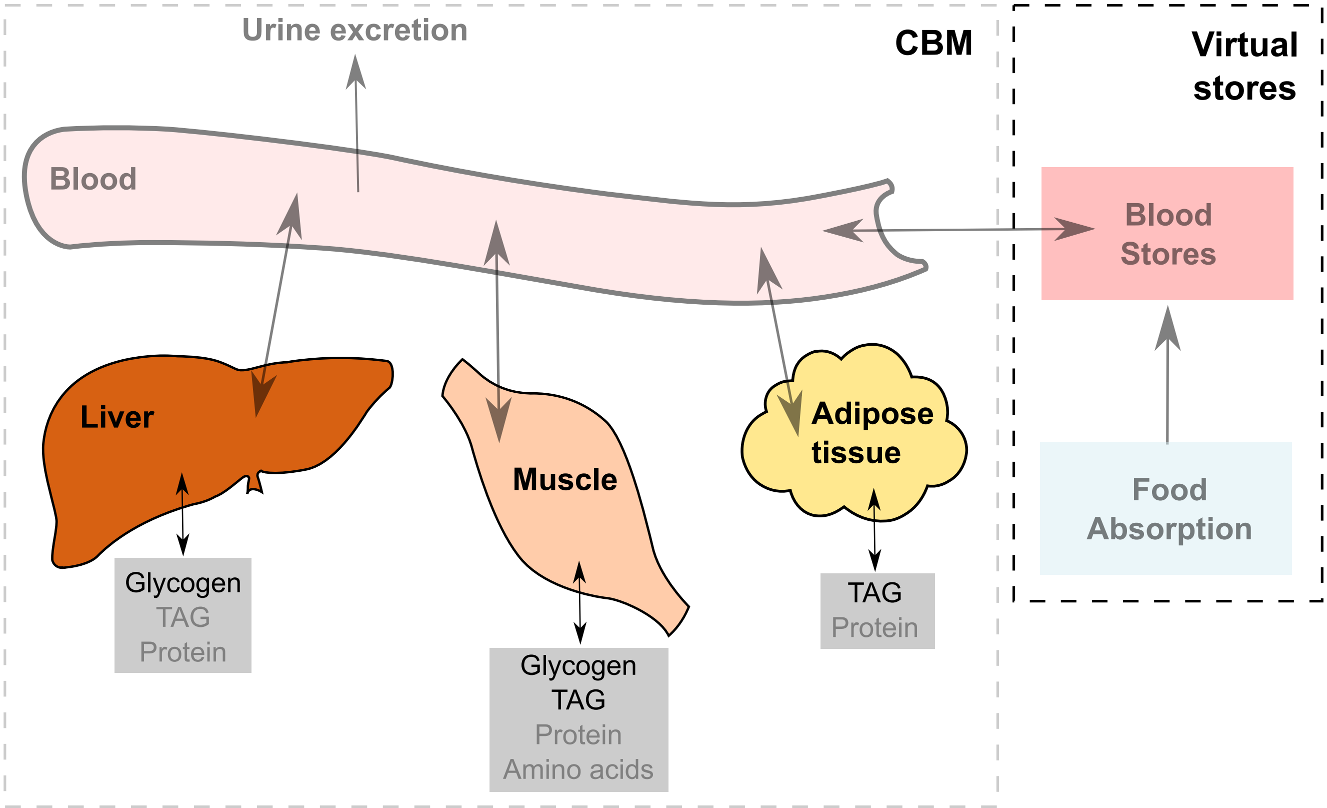

Supplementary Figure 13. Contribution of the energy stores to the objective value. The degradation, and production of energy sources was balanced within the different tissues by using the coefficients on Supplementary Table 8. A negative coefficient was used to encourage the model to store energy, which could be used when external energy was not provided (i.e. in fasting conditions). On the one hand, the storage of energy sources was encouraged, and the encouraging factor increased with an increased absolute α_{store} value. On the other hand, the usage of energy sources from the stores was penalized, and the penalization factor increased with the increase in the β_{store} value. This results in the model aiming to first store glycogen in the liver, and in the muscle tissues. If energy would be in excess then the energy could be stored in the adipose tissue. Additionally, the adipose tissue, and the liver were the main providers of energy in the fasting conditions. Because of the penalization factor, the muscle would only become the main supplier of energy, in case of a high demand of energy (i.e. during exercise). To prevent the usage of one energy store, to refill another energy store, the coefficients in Supplementary Table 8 were selected in the following way: the absolute β_{store} values had to be larger than the absolute values of the α_{store}. Abbreviations: TAG=Triacylglycerol.

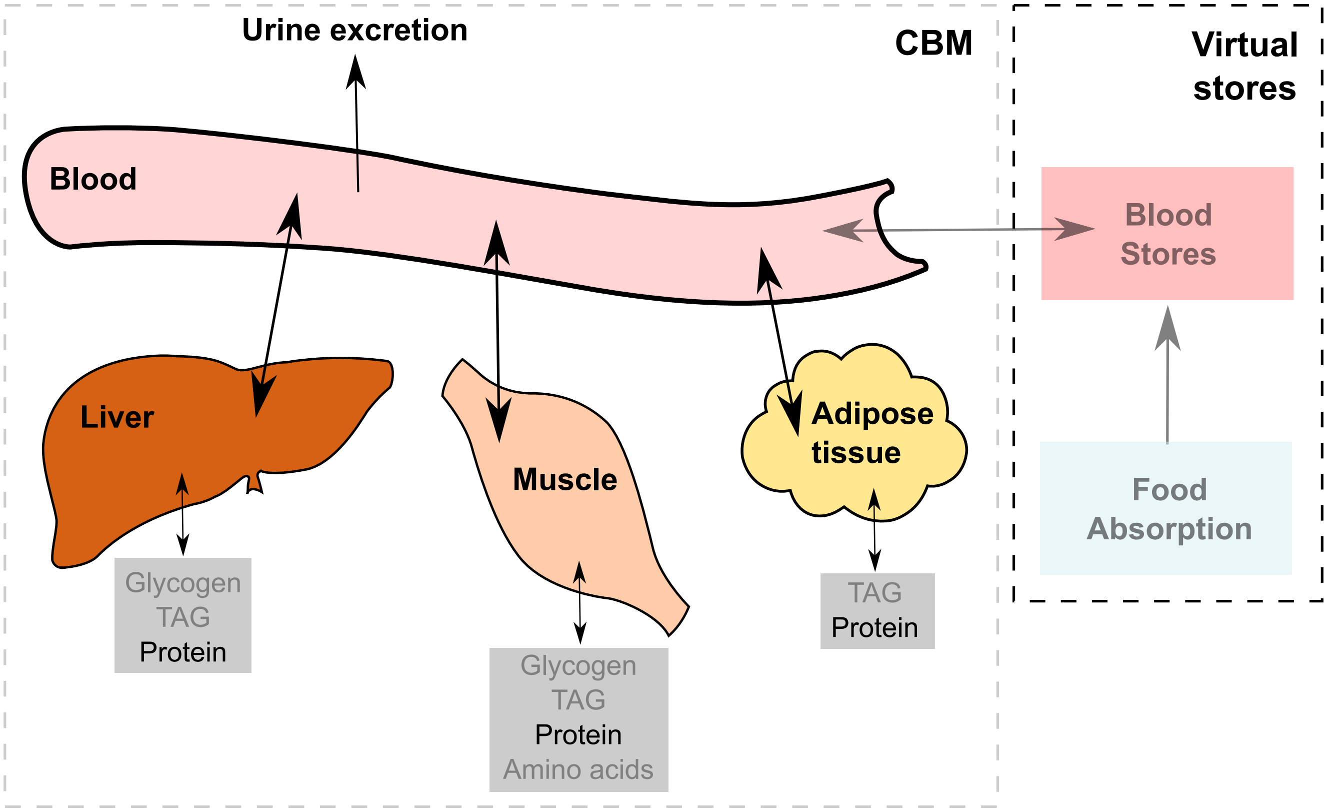

Supplementary Figure 14. Flexible constraints on reaction fluxes derived from literature. Flexible constraints were used to constrain the model, while still allowing reactions to carry a larger, or smaller flux than the flux derived from literature. Urine excretion reactions, and tissue exchange reactions were constrained using the healthy flux bounds, when available. Transport reactions (R_{CS}), which were added to the tissue models, were constrained to have a zero flux. As these reactions were manually added, their flux was minimized, forcing the model to only allow flux through these reactions if really necessary. The protein degradation (R_{PD}) was minimized, by constraining the protein degradation reactions to carry a null flux. Simultaneously, in case protein degradation was necessary, the sum of the reaction fluxes could not be larger than the maximum degradation rate. The coefficients in Table 8 were used to balance the protein degradation fluxes within the tissues. A smaller coefficient allowed a lower penalization of protein degradation. Therefore by using a coefficient of 0.1 for the protein degradation in the muscle, the muscle became the main protein source during the fasting condition. Abbreviations: TAG=Triacylglycerol.

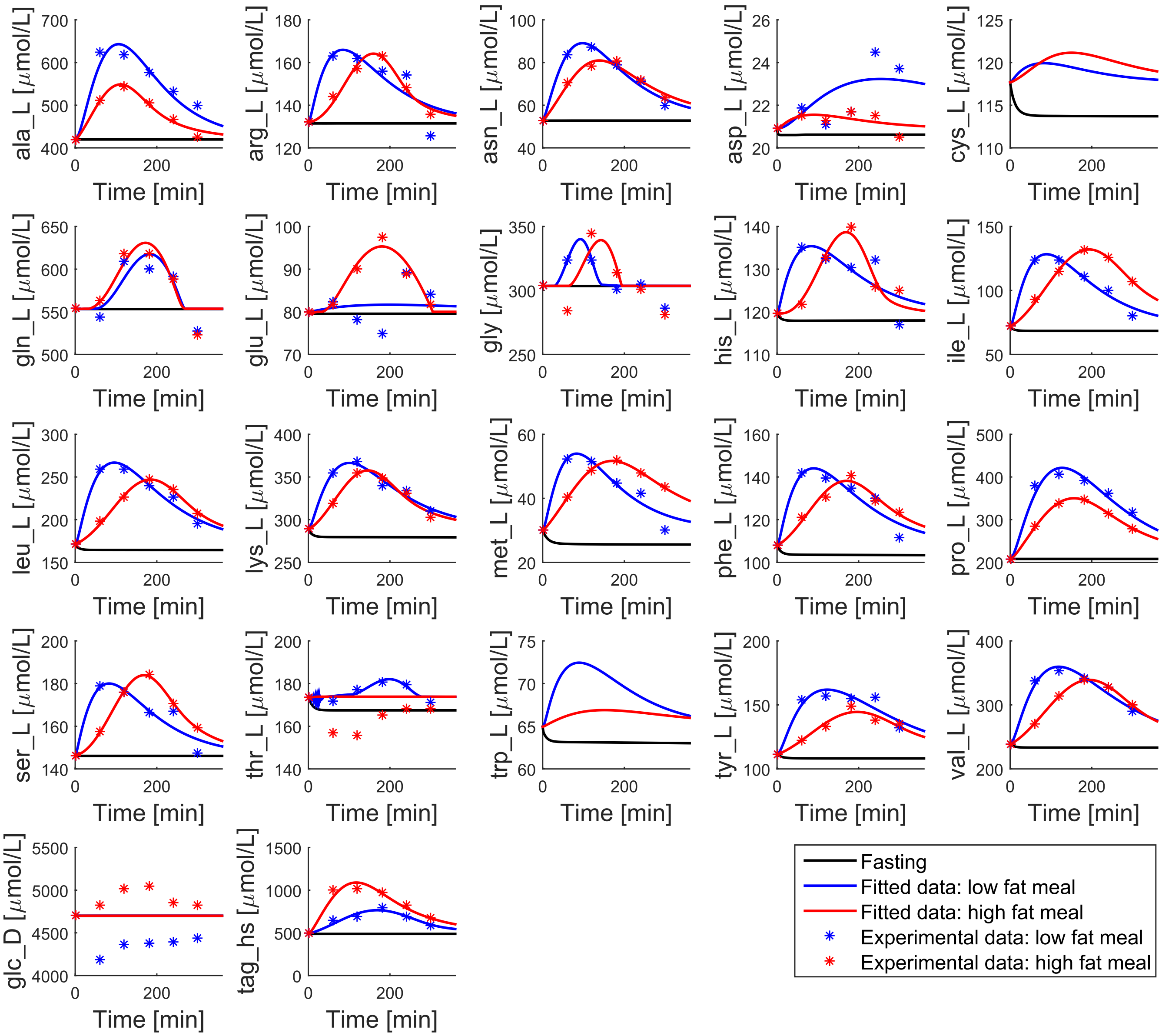

Supplementary Figure 15. Blood metabolite concentrations fitted to the mean blood metabolite concentration data from Milan et al.[1], after the ingestion of a low and a high fat meal. Additionally, the blood concentrations predicted in the fasting condition are shown. No data was available for cysteine, and tryptophan, therefore the parameters have been selected as mentioned in the Methods section.

Supplementary Table 1. Model prediction of all known biomarkers for Arginase deficiency. Known biomarkers for Arginase deficiency were collected from different databases. Conditions highlighted in blue showed good agreement with reported data. References for the different databases: Ramedis[2]; IEMbase[3]; HMDB[4–7]; SMPDB[8,9]; Metagene[10]. Symbols: N=Normal; ↗=Increased; ↘=Decreased.

Metabolite	Fluid	Ramedis Level	IEMbase Level	HMDB Level	SMPDB Level	Metagene Level	Model prediction
5-Oxoproline	Urine	↗					↗
Ammonia	Serum/plasma	N or ↗	↗	↗	↗	↗	↗
Ammonia	Urine						↗
Arginine	Serum/plasma	↗	↗		↗	↗	N
Arginine	Urine	N or ↗					↗
Carnitine, free	Blood	N					N
Citrulline	Serum/plasma		N or ↗				↗
Citrulline	Urine	↗					↗
Creatine	Serum/plasma				↗		N
Creatine	Urine						↗
Cystine	Urine	N or ↗					N
Glutamine	Serum/plasma		↗				N
Glutamine	Urine						↗
Ornithine	Serum/plasma	N or ↘		↘			↗
Ornithine	Urine						↗
Orotic acid	Serum/plasma			↗			↗
Orotic acid	Urine	↗	N or ↗	↗	↗	↗	↗
Uracil	Serum/plasma						↗
Uracil	Urine	↗		↗	↗		↗
Urea	Serum/plasma		N or ↘				N
Urea	Urine						↘

Supplementary Table 2. List of cel files used for the reconstruction of the tissue-specific models. Data was collected from GEO database.

Liver	Skeletal muscle	Subcutaneous adipose tissue
GSM138595.CEL	GSM465275.CEL.gz	GSM176028.CEL.gz
GSM138596.CEL	GSM465277.CEL.gz	GSM176119.CEL.gz
GSM155919.CEL	GSM465279.CEL.gz	GSM176121.CEL.gz
GSM155926.CEL	GSM465284.CEL.gz	GSM691122.CEL.gz
GSM155927.CEL	GSM465289.CEL.gz	GSM691123.CEL.gz
GSM155928.CEL	GSM465292.CEL.gz	GSM691124.CEL.gz
GSM155947.CEL	GSM465294.CEL.gz	GSM691130.CEL.gz
GSM155948.CEL	GSM465295.CEL.gz	GSM691131.CEL.gz
GSM155961.CEL	GSM465297.CEL.gz	GSM691134.CEL.gz
GSM155964.CEL	GSM465302.CEL.gz	GSM691135.CEL.gz
GSM155988.CEL	GSM465303.CEL.gz	GSM691143.CEL.gz
GSM155989.CEL	GSM465309.CEL.gz	GSM691147.CEL.gz
GSM176332.CEL	GSM465310.CEL.gz	GSM691150.CEL.gz
GSM176333.CEL	GSM465313.CEL.gz	GSM691153.CEL.gz
GSM176334.CEL	GSM465316.CEL.gz	GSM691154.CEL.gz
GSM176335.CEL	GSM465320.CEL.gz	
GSM279063.CEL	GSM465323.CEL.gz	
GSM279064.CEL	GSM465329.CEL.gz	
GSM279065.CEL	GSM465332.CEL.gz	
GSM572800.cel	GSM465339.CEL.gz	
GSM572801.cel	GSM465346.CEL.gz	
GSM572802.cel	GSM465351.CEL.gz	
GSM572803.cel	GSM465353.CEL.gz	
GSM572804.cel	GSM465354.CEL.gz	
GSM572805.cel	GSM465355.CEL.gz	
GSM572806.cel	GSM465357.CEL.gz	
GSM80728.CEL	GSM465359.CEL.gz	
GSM80729.CEL	GSM465361.CEL.gz	
GSM80730.CEL	GSM465363.CEL.gz	
GSM80739.CEL	GSM465364.CEL.gz	
	GSM465365.CEL.gz	
	GSM465366.CEL.gz	
	GSM465368.CEL.gz	
	GSM465369.CEL.gz	
	GSM465371.CEL.gz	
	GSM465372.CEL.gz	
	GSM465373.CEL.gz	
	GSM465374.CEL.gz	
	GSM465376.CEL.gz	
	GSM465379.CEL.gz	
	GSM465381.CEL.gz	
	GSM465384.CEL.gz	
	GSM465385.CEL.gz	
	GSM465386.CEL.gz	
	GSM465390.CEL.gz	
	GSM465391.CEL.gz	

Supplementary Table 3. Weight and density of the different tissues. These values were used to convert the literature fluxes to total tissue flux per minute.

**Tissue**	**Weight** (*grams*)	**Density** (*grams/milliliter*)
Liver	2360	
Muscle	32000	1.1
Adipose tissue	12430	

Supplementary Table 4. Initial basal amounts and ranges for the internal energy tissue stores. Abbreviation: inf=infinity.

**Tissue**	**Store**	**Basal Amount** (*grams*)	**Molecular Weight** (*grams/mol*)	**Basal Amount** (μmol)	**Allowed range** (μmol)
Liver	Glycogen	100	1800	$5.5556 \cdot 10^4$	$0 - 8.3333 \cdot 10^4$
Liver	Protein	429.52	134	$3.2054 \cdot 10^6$	$0 - 3.3656 \cdot 10^6$
Muscle	Glycogen	380	1800	$2.1111 \cdot 10^5$	$0 - 2.7778 \cdot 10^5$
Muscle	TAG	300	879	$3.4130 \cdot 10^5$	$0 - \text{inf}$
Muscle	Protein	5760	134	$4.2985 \cdot 10^7$	$0 - 4.5134 \cdot 10^7$
Adipose tissue	TAG	11187	879	$1.2727 \cdot 10^7$	$0 - \text{inf}$
Adipose tissue	Protein	261	134	$1.9478 \cdot 10^6$	$0 - 2.0451 \cdot 10^6$

Supplementary Table 5. ATP demand estimation. In red are denoted ATP consuming processes that were not accounted for in the model and are used for the estimation of the standard metabolic rate (SMR). The weight of the tissues were extracted from the Open Systems Pharmacology Suite (OSPS) 7.1, assuming an European Man of 30 years old, 70 kg, and 176 cm.

	**Liver**	**Muscle**	**Adipose tissue**	**Units**	**References**
Weight	2360	32000	12430	g	OSPS
SMR	200	13	4.5	kcal/kg/day	Wang et al.[11]
Estimation of total ATP demand	44901	39574	5321	µmol/min	
Non mitochondrial oxygen consumption	20	14		%	Rolfe & Brown[12]
Body Oxygen Use	17	20		%	
Protein synthesis	24.00	17.00		%	Rolfe & Brown[12]
Na^+/K^+	20.00	20.00		%	
Ca^{2+}	2.00	6.00		%	
Gluconeogenesis	5.00			%	
Urea synthesis	12.00			%	
Actinomyosin				%	
Substrate cycling	26.00	7.50		%	
RNA turnover	1.80	1.80		%	
Proton leak	4.42	10.40		%	
Non mitochondrial	3.40	2.80		%	
Estimation of % of SMR not included in model	53.20	38.10	38.00	%	
Estimation of ATP consumption not included in model	23887	15078	2022	µmol/min	

Supplementary Table 6. Protein turnover estimation. A whole-body protein turnover rate of 0.9 g/kg/day[13] was converted to µmol/min in several steps. The average molecular weight of all amino acids was calculated. This value allowed to convert the protein grams to µmol, thus enabling the determination of the whole-body protein turnover rate of an 70 kg male individual. Tissue protein turnover rates were calculated for each tissue from the whole-body protein turnover rate. For the liver and the adipose tissue, this calculation was based on the tissue weights reported in the Supplementary Table 12. The muscle protein turnover was considered to be 30% of the whole-body one[14]. The amino acids (AA) coefficients were then normalized to the tissue protein turnover rate in several steps. For each AA *i*, in each tissue, this was achieved by using Supplementary Equation 1. The calculation of the total NH4 produced was performed in several steps. For each AA *i*, the number of nitrogen was collected. This value was then multiplied by the respective adjusted AA coefficient and the sum was calculated. The bounds of the protein turnover reaction for each tissue were fixed to 1 μmol/min. Abbreviations: AA=amino acid; PTR=protein turnover rate.

Tissue	Reaction
Liver	0.963647625 ala_L[c] + 0.530440165 arg_L[c] + 0.502634086 asn_L[c] + 0.535399133 asp_L[c] + 0.235908598 cys_L[c] + 0.635111322 gln_L[c] + 0.678680027 glu_L[c] + 1.101083936 gly[c] + 0.235023091 his_L[c] + 0.492007572 ile_L[c] + 0.918484943 leu_L[c] + 0.807083696 lys_L[c] + 0.182598887 met_L[c] + 0.329953242 phe_L[c] + 0.52548109 pro_L[c] + 0.583041406 ser_L[c] + 0.583041406 thr_L[c] + 0.060765911 trp_L[c] + 0.239450734 tyr_L[c] + 0.646269161 val_L[c] => 14.85306772 nh4[c]
Muscle	8.1520 ala_L[c] + 4.0760 arg_L[c] + 5.1634 asn_L[c] + 5.1634 asp_L[c] + 1.9169 cys_L[c] + 6.4202 gln_L[c] + 6.4202 glu_L[c] + 9.1033 gly[c] + 2.3095 his_L[c] + 4.0080 ile_L[c] + 7.8811 leu_L[c] + 7.6090 lys_L[c] + 1.6984 met_L[c] + 3.0567 phe_L[c] + 4.9593 pro_L[c] + 4.7552 ser_L[c] + 5.0953 thr_L[c] + 0.5132 trp_L[c] + 1.9706 tyr_L[c] + 5.7064 val_L[c] => 132.5310 nh4[c]
Adipose tissue	4.949695363 ala_L[c] + 2.354682388 arg_L[c] + 2.648156343 asn_L[c] + 2.648156343 asp_L[c] + 0.985543965 cys_L[c] + 3.251270483 gln_L[c] + 3.251270483 glu_L[c] + 7.535460587 gly[c] + 1.083068215 his_L[c] + 2.41938891 ile_L[c] + 4.797183741 leu_L[c] + 3.14035294 lys_L[c] + 0.732984719 met_L[c] + 2.198704593 phe_L[c] + 3.761948717 pro_L[c] + 3.126488247 ser_L[c] + 2.696682768 thr_L[c] + 0.31171989 trp_L[c] + 1.469421747 tyr_L[c] + 3.447691588 val_L[c] => 75.39155528 nh4[c]

The normalization of the amino acids coefficients involved in the protein turnover reaction was performed using Supplementary Equation 1. These coefficients were extracted from the biomass reactions available in Bordbar et al.[15].

$$AdjustedAA_{coefficient,\ i} = \frac{AA_{coefficient,\ i,\ tissue} \cdot PTR_{tissue}}{\sum AA_{coefficient,\ tissue}} \qquad (1)$$

Supplementary Table 7. Composition of the amino acids stores in the muscle tissue. Note: all amino acids stores are in μmol.

Reaction Name	Average Amount	Minimal Amount	Maximal Amount	Reference
Muscle_ala_L_store	28508	24637	32010	Lundholm et al.[16]
Muscle_arg_L_store	58737	37049	65557	
Muscle_asn_L_store	5038	2335	5837	
Muscle_asp_L_store	6636	3994	12104	
Muscle_gln_L_store	141189	110162	160051	
Muscle_glu_L_store	32502	20398	37970	
Muscle_gly_store	13056	11182	14162	
Muscle_his_L_store	3748	3133	4485	
Muscle_ile_L_store	1782	1536	5161	
Muscle_leu_L_store	3502	3195	8417	
Muscle_lys_L_store	6328	3502	8909	
Muscle_met_L_store	614	492	1597	
Muscle_phe_L_store	1106	922	4792	
Muscle_ser_L_store	9032	7066	13455	
Muscle_thr_L_store	13056	11182	14162	
Muscle_tyr_L_store	58737	37049	65557	
Muscle_val_L_store	4547	4116	11428	

Supplementary Table 8. Values of storage (α_{store}) and release (β_{store}) coefficients. EXP/exp represents the metabolite production and its storage. IMP/imp represents the metabolite degradation from the store. The β_{store} coefficients for the energy stores usage were added to the quadratic objective, therefore ensuring a stronger penalization of large active fluxes. Abbreviations: QP=Quadratic part of the objective; LP=Linear part of the objective.

Coefficient	Tissue	Source	Reaction Name	Coefficient value	LP or QP
α_{store}	Liver	Glycogen	Hep_GLYGN1_EXP	-0.001	LP
	Muscle	Glycogen	Muscle_GLYGN1_EXP	-0.001	LP
	Adipose tissue	TAG	Fat_tag_stores_exp	-0.0001	LP
	Muscle	TAG	Muscle_tag_stores_exp	-0.0001	LP
	Liver	Protein	Hep_protein_production	1	QP
	Muscle	Protein	Muscle_protein_production	1	QP
	Adipose tissue	Protein	Fat_protein_production	1	QP
β_{store}	Liver	Glycogen	Hep_GLYGN2_IMP	1.5	QP
	Muscle	Glycogen	Muscle_GLYGN2_IMP	15	QP
	Adipose tissue	TAG	Fat_tag_stores_imp	0.4	QP
	Muscle	TAG	Muscle_tag_stores_imp	15	QP
	Liver	Protein	Hep_protein_degradation	0.9	QP
	Muscle	Protein	Muscle_protein_degradation	0.1	QP
	Adipose tissue	Protein	Fat_protein_degradation	1	QP

Supplementary Table 9. List of coefficients of specific reactions integrated in the quadratic part of the objective function.

Coefficient	Tissue	Reaction Name	Coefficient value
τ	Liver	Hep_glygn_stores	75
	Muscle	Muscle_O_2	0.01
	All tissues	CS as detailed in Table 3	10
		All reactions	0.001

Supplementary Table 10. List of metabolites amounts present in the low and high fat meal and their respective fitted parameters.

Metabolite	Low fat meal [μmol]	High fat meal [μmol]	Low fat meal			High fat meal		
			**Tmax**	**vmax**	**Km**	**Tmax**	**vmax**	**Km**
alanine	20241	25431	80.53	325.47	999.82	89.63	124.58	106.89
arginine	13579	14438	79.43	499.99	443.63	131.75	33.87	8.44
asparagine	15098	16790	90.87	175.64	138.84	128.78	70.41	38.16
aspartate	14986	16667	199.61	92.24	818.63	84.76	626.15	689.19
cysteine	2004	10720	80.2	1000	546.99	147.75	55.5	14.22
glutamine	35300	30810	121.38	33.6	1.19	110.48	31.93	2.21
glutamate	35063	30603	190.1	171.62	242.02	85.29	1.83	0
glycine	19919	24218	71.93	75.2	4.59	106.47	56.97	0.95
histidine	8503	8612	80.05	1000	752.77	133.18	18.04	2.59
isoleucine	18835	17913	82.8	727.52	778.04	158.4	39.6	25.52
leucine	32430	28669	88.99	914.9	1000	155.17	63.29	29.82
lysine	22852	22218	92.05	386.19	458.44	122.18	66.83	35.3
methionine	7834	7593	76.81	201.11	193.69	150.7	24.76	22.19
phenylalanine	15299	13601	84.75	1000	931.31	148.82	34.62	16.89
proline	38324	27029	109.91	473.52	999.61	128.61	107.86	157.37
serine	26389	24539	80.2	1000	546.99	147.75	55.5	14.22
threonine	14651	15508	169.22	23.23	0.71	91.46	859.73	43.31
trypyophan	3335	2996	84.75	1000	931.31	148.82	34.62	16.89
tyrosine	11946	9262	111.77	510.61	985.17	162	21.21	16.83
valine	26348	23611	106.31	535.3	990.86	154.42	54.07	44.34
triacylglycerol	21897	74481	117.82	88.96	113.36	97.68	699.62	999.78
glucose	411553	527304	195.72	984.06	7.01	200	1000	25.9

References

1. Milan, A. *et al.* Older adults have delayed amino acid absorption after a high protein mixed breakfast meal. *The journal nutrition, health & aging* **19**, 839–845 (2015).

2. Ramedis database. http://www.ramedis.de.

3. Lee, J. J., Wasserman, W. W., Hoffmann, G. F., van Karnebeek, C. D. & Blau, N. Knowledge base and mini-expert platform for the diagnosis of inborn errors of metabolism. *Genet. Medicine* **20**, 151 (2018).

4. Wishart, D. S. *et al.* Hmdb: the human metabolome database. *Nucleic acids research* **35**, D521–D526 (2007).

5. Wishart, D. S. *et al.* Hmdb: a knowledgebase for the human metabolome. *Nucleic acids research* **37**, D603–D610 (2008).

6. Wishart, D. S. *et al.* Hmdb 3.0– the human metabolome database in 2013. *Nucleic acids research* **41**, D801–D807 (2012).

7. Wishart, D. S. *et al.* Hmdb 4.0: the human metabolome database for 2018. *Nucleic acids research* **46**, D608–D617 (2017).

8. Jewison, T. *et al.* Smpdb 2.0: big improvements to the small molecule pathway database. *Nucleic acids research* **42**, D478–D484 (2013).

9. Frolkis, A. *et al.* Smpdb: the small molecule pathway database. *Nucleic acids research* **38**, D480–D487 (2009).

10. Metagene database. http://www.metagene.de.

11. Wang, Z. *et al.* Specific metabolic rates of major organs and tissues across adulthood: evaluation by mechanistic model of resting energy expenditure–. *The Am. journal clinical nutrition* **92**, 1369–1377 (2010).

12. Rolfe, D. & Brown, G. C. Cellular energy utilization and molecular origin of standard metabolic rate in mammals. *Physiol. reviews* **77**, 731–758 (1997).

13. Poortmans, J., Carpentier, A., Pereira-Lancha, L. & Lancha Jr, A. Protein turnover, amino acid requirements and recommendations for athletes and active populations. *Braz. J. Med. Biol. Res.* **45**, 875–890 (2012).

14. Welle, S. *Human protein metabolism* (Springer Science & Business Media, 2012).

15. Bordbar, A. *et al.* A multi-tissue type genome-scale metabolic network for analysis of whole-body systems physiology. *BMC systems biology* **5**, 180 (2011).

16. Lundholm, K. *et al.* Transport kinetics of amino acids across the resting human leg. *The J. clinical investigation* **80**, 763–771 (1987).
